# Nacre and Nacre-Inspired Materials: Historical Background, Definition, Fabrication Techniques and Gaps

**DOI:** 10.3390/biomimetics11020148

**Published:** 2026-02-16

**Authors:** Naim Sedira, João Castro-Gomes, Jorge Pinto, Pengkou Hou, Sandra Pereira

**Affiliations:** 1 C-MADE, The Centre of Materials and Civil Engineering for Sustainability, Universidade da Beira Interior (UBI), 6201-001 Covilhã, Portugal; jpcg@ubi.pt (J.C.-G.); tiago@utad.pt (J.P.); spereira@utad.pt (S.P.); 2 Department of Civil Engineering and Architecture, University of Beira Interior (UBI), 6200-358 Covilhã, Portugal; 3 Department of Engineering, School of Sciences and Technologies, University of Trás-os-Montes e Alto Douro (UTAD), 5000-801 Vila Real, Portugal; 4 Shandong Provincial Key Laboratory of Green and Intelligent Building Materials, University of Jinan, Jinan 250022, China; mse_houpk@ujn.edu.cn

**Keywords:** nacre, nacre-inspired, structure, biomimetic, composites, biomineralisation

## Abstract

From Palaeolithic ornaments to modern biomimetics, the use of nacre and shells has evolved. Initially utilised for jewellery and tools, they now inspire the development of advanced materials. This paper reviews the current knowledge on nacre’s composition, focusing on the highly regulated biomineralisation process wherein amorphous calcium carbonate (ACC) transforms into crystalline aragonite. It examines the important role of the organic matrix (specifically soluble, insoluble, and acidic proteins) in controlling crystal nucleation, growth, and polymorph selection. Scientists study natural nacre formation to create nacre-inspired composites for various applications. Charles Hatchett’s in 1799 shell categorisation, Sorby and Sowerby’s 19th-century microscopy, Taylor, Beedham, Bøggild, and Currey’s mid-20th-century research on bivalve structures, and mechanical property investigations in the 1970s are some of the major developments. The hierarchical structure, cooperative plastic deformation, surface asperities, organic–inorganic interactions, and interphase in such complex composite materials give rise to impressive mechanical properties. In the early 2000s, with the emergence of biomimetics, inspired by nacre, several macroscopic structural materials with uniform micro- and nanoscale architectures have been synthesised in recent decades, and their mechanical properties and potential applications have been explored. Modern nacre-inspired fabrication utilises 3D printing for precision, freeze casting for sustainability, and mineralisation for scalability. Techniques like layer-by-layer assembly and nanomaterial integration enhance mechanical performance through advanced interfacial engineering.

## 1. Introduction

Pearls and nacre, also known as mother of pearl, have been valued for their beauty since ancient times and have been the subject of scientific study for at least 150 years [1]. At the same time, natural nacre is a biomineral with a complex structure composed of aragonite nanograins surrounded by an organic matrix. The organic matrix of nacre contributes substantially to initiating and guiding the biomineralisation process [2]. Nacre possesses extraordinary mechanical properties due to its hierarchical structure, which arises from the assembly of different-sized building blocks during its growth [3,4]. Nacre is a hierarchical natural composite comprising calcium carbonate (CaCO_3_) platelets bonded by a biopolymer. Its remarkable mechanical properties stem from this hierarchical structure, which results from assembling differently sized building blocks during growth. While predominantly mineral-based, nacre exhibits a toughness significantly higher than pure mineral materials. This toughness is partly due to its brick-and-mortar arrangement of aragonite platelets, which provides an architecture distinct from that of traditional engineering materials. Several mechanisms, such as crack deflection, crack bridging, friction between nanoasperities, and plate pullout, enhance its toughness by improving energy dissipation [4,5,6,7,8,9]. Furthermore, nacre’s unique brick-and-mortar architecture confers anisotropy on its mechanical properties. However, its weakest direction experiences tension perpendicular to the orientation of the mineral bricks, while its strongest direction involves compression perpendicular to this orientation [10,11]. Nacre has been studied for its potential applications in bone regeneration and repair, as it is osteoinductive, osteoconductive, biocompatible, and biodegradable [12]. Additionally, nacre has shown interesting properties for human health, with studies investigating its activity on bone mineralisation and its potential use in skin care [13]. Understanding the formation of nacre is of interest not only for biology and geology but also for materials science, as it could lead to the development of new layered materials with tuneable properties [12].

Understanding the properties of nacre can inform biomimetic research aimed at emulating natural designs for technological innovation. In recent years, systematic studies have been conducted to better understand and apply more sophisticated capabilities in this field, which is increasingly referred to as biomimetics [14]. “Biomimetics” originates from the Ancient Greek word “biomimesis”, which combines bios (meaning life) and mimesis (meaning imitation) to signify the mimicking of living nature. This term was coined in 1957 by polymath Otto Schmitt to describe the emulation of biological processes for technological innovation. Fundamentally, biomimetics involves studying the formation, structure, and function of biological substances and mechanisms to synthesise analogous products and solutions through artificial means [15,16,17].

In materials science and engineering, the groundwork is laid on the fundamental necessity of durable, resilient materials capable of withstanding significant stress and strain across diverse operating conditions and environments [18,19]. These materials contribute significantly to sectors such as aerospace, automotive, and infrastructure [20]. Nacre-inspired materials have garnered significant attention in research owing to their exceptional mechanical properties. Several studies in the last decade have investigated the design and enhancement of bioinspired composites based on the hierarchical structures present in nacre shells [21]. These composites aim to balance multiple material properties, such as strength, toughness, and specific volume. Furthermore, nacre-inspired structures in nanocomposites have been explored to augment their toughness and strength [22]. Other studies have focused on developing nacre-inspired fibres and films with high strength and toughness by incorporating tough interlayer entanglement. These materials show promise for various applications, including wearable energy storage devices and mechanochromic sensors [23,24,25]. While much of the existing literature has examined the chemistry, mechanical properties, and synthesis of nacre and nacre-inspired materials, this paper presents a comprehensive, chronological, and forward-looking overview. This review reconstructs the historical evolution of nacre research from its earliest anthropogenic uses in the Palaeolithic and Neolithic eras to the foundational scientific classifications of Charles Hatchett and Henry Clifton Sorby in the 18th and 19th centuries. Furthermore, this work bridges the gap between traditional structural mechanics and emerging technologies by integrating recent advances in machine learning-driven design, smart self-sensing capabilities, and energy storage applications, such as solid-state batteries. Significantly, rather than being limited to a retrospective, this review includes a dedicated analysis of current research gaps—ranging from 3D printing alignment limitations to multifunctionality trade-offs—and presents a strategic roadmap for overcoming the specific engineering hurdles that currently limit the field.

## 2. Historical Background of Nacre-Inspired Materials

**Figure 1:**
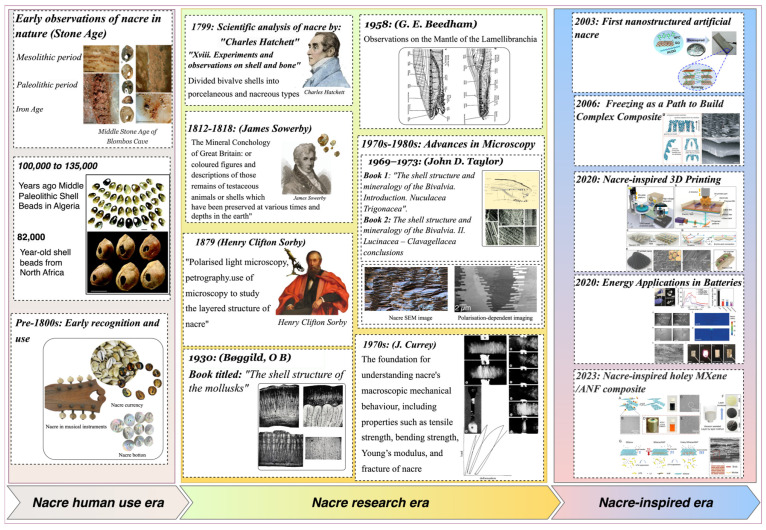
Chronological evolution from the early Stone Age to the development of nacre-inspired synthetic materials.

Figure 1 presents a schematic timeline illustrating the evolution of nacre, tracing its utilisation by humans as a decoration to the development of novel bioinspired advanced materials. The transition from natural nacre to various forms of nacre-inspired synthetic materials has necessitated decades of extensive scientific research and innovation. For over a century, biologists have studied the structure of nacre, but in recent years this topic has attracted increasing attention. Many researchers have investigated the physical and mechanical properties of nacre or tried to synthesise its structure. Various techniques have been used to investigate the inorganic and bioorganic phases, including X-ray diffraction, spectroscopy, and microscopy. The investigations have been multidisciplinary, with scientists from multiple fields collaborating in these studies. The authors of this report integrate work from synthetic and biological sciences, relating findings to practical applications, including stable coatings with underwater superoleophobicity [26], pearls, and intelligent polymeric coatings [27]. Although preliminary methods had been developed long ago, in recent years, ceramic heritage researchers have significantly improved control over relevant parameters, enabling the synthesis of new materials that successfully replicate the complex structure of nacre.

### 2.1. Early Observations of Nacre in Nature (Stone Age)

Humans have long been captivated by nature’s ability to create aesthetically beautiful and functional materials from simple resources. The human use and admiration of both abalone and mother of pearl extend throughout our history. Nacre has been appreciated for thousands of years through its use in jewellery, utensils, and inlay work [28,29,30,31]. The use of nacre dates back to the Stone Age, which is usually divided into three separate periods.

#### 2.1.1. Palaeolithic Period

Middle Palaeolithic shell beads from 100,000 to 135,000 years ago were found in Algeria [32], and 82,000-year-old shell beads, specifically perforated Nassarius gibbosulus, were found at Grotte des Pigeons (Taforalt, Morocco). These are among the earliest known examples of personal ornaments [33]. These beads indicate the use of symbols to convey social messages, a hallmark of modern human behaviour [32,33,34,35].

#### 2.1.2. Mesolithic Period

In the Middle Stone Age, evidence shows that humans used marine resources, including nacre, in South Africa [36]. This period documents the earliest systematic gathering of marine resources, including shellfish, which likely included nacreous shells [33,37].

#### 2.1.3. Neolithic Period

During the Neolithic period (c. 6500–3300 BCE), humans used nacre to make tools and personal adornments. This period saw advanced seafaring and the exploitation of marine resources, including the use of large marine mollusc shells for various tools [38].

#### 2.1.4. Iron Age

The Nafūn shell midden in Oman provides evidence that, during the Iron Age II period, people utilised marine resources, including nacre, for economic and subsistence purposes [39]. Moreover, M. Mărgărit [40] investigates the ornaments used by human societies in Europe along the Lower Danube during the 6th–5th millennia BC. Ornaments from 22 settlements and 19 cemeteries were studied in this research, including raw materials, their origin, production processes, and use as ornaments. This period evidences the cultural role of marine resources in long-distance trade and contacts.

### 2.2. Pre-1800s: Early Recognition and Use

During the 1600–1700s, nacre was widely used to create decorative items, art, and musical instruments. More particularly, it was used during the late Ottoman and British Mandate periods in Palestine in producing religious souvenirs and other decorative items. During that period, the chemical properties of nacre—as well as its iridescence and strength—made it suitable for artistic and decorative use. The cultural and societal implications of its use were evident in the production and trade of religious souvenirs and ornamental items: nacre was imported from Europe and used in the manufacture of religious souvenirs and ornamental items in Bethlehem and Jerusalem, and the finished products were transported back to Europe and elsewhere. Moreover, paper, parchment, and reused materials were in common use for the making and repair of musical instruments during the 1600–1700s, further underscoring the manifold uses of materials in artistic and decorative contexts [28,41]. In Korea, artisans before the mid-Joseon period (mid-1500s to mid-1600s) manually shaved away the layers, but later began using a turning wheel to sand the shell’s outer and middle layers [42,43]. Although the process of nacre extraction and shaping is the same between civilisations (removing the nacre from the shell and then shaping it into a desired form), their methods and the species of shells they choose are different.

Nacre has been used by humans for centuries in jewellery, buttons, and other ornaments. The fascination with nacre has recently expanded into a scientifically driven interest in understanding the structures responsible for its colour and mechanical properties, following initial research into similar structures found in ceramics and crystals. As a chemist, C. Hatchett (1765–1847), whose education was self-directed, published studies concerning material composition, especially the chemical structure of hard tissues, such as shells, bone, and teeth. He made an important contribution to the knowledge of the structure of bivalve shells, which was that of Hatchett in 1799, who divided them into porcelaneous and nacreous types [44]. This classification provided a framework for later studies of bivalve shell structure. Subsequent studies have shown that both the composition and architecture of bivalve shells are complex and varied. The decades following Hatchett’s work saw the recognition of additional shell types besides the original porcelaneous and nacreous.

### 2.3. 1800s: Early Microscopic Observations

**Figure 2:**
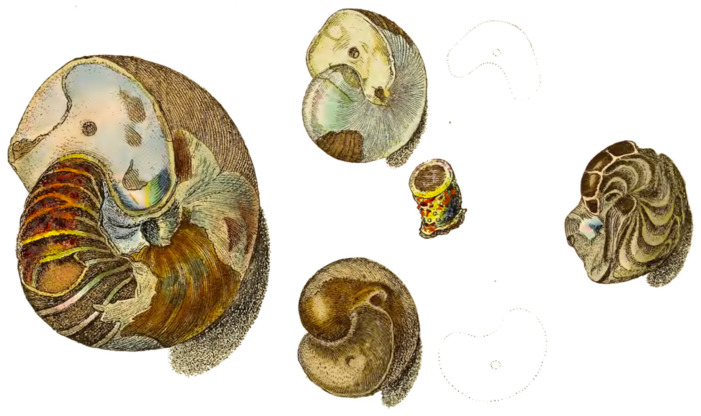
Great Clay stratum recently exposed at Highgate, with some of the brownish outer coat remaining [45].

In 1812, James Sowerby published a book titled *The mineral conchology of Great Britain; or coloured figures and descriptions of those remains of testaceous animals or shells, which have been preserved at various times and depths in the earth* [45]. Among the many studies in his book, Sowerby described a specimen from the Great Clay stratum recently exposed at Highgate (Figure 2). This specimen still retained some of its brownish outer coat. The shell was broken at the aperture, which was sealed by a pearly concave septum revealing the *siphunculus* opening. The remaining parts of the shell were also pearly and iridescent. Sowerby remarked that the broad undulations of the *septae*, with their shining brown deposits of calcium carbonate in between, gave the appearance of a lobster’s tail. Another section showed the exposed chambers, the first of which was lined with a yellowish, more waxy lime of calcium carbonate and exhibited part of the *siphunculus*.

Henry Clifton Sorby was one of the pioneers in the microscopic investigation of nacre. He was the first to observe the layered structure of this natural composite material. Sorby developed the method of preparing thin, polished sections of rocks and minerals for study under polarised light microscopes. These techniques enabled the close inspection of microstructures, which were innovative at the time and remain widely used today with minimal modifications. Sorby also researched microscopic metal surfaces and fractures [46,47,48]. In 1879, he wrote an article in *Nature* in which he mentioned that the original composition of the shell has a great influence on fossil preservation. Calcite shells resist and preserve their structure, whereas aragonite shells dissolve or are changed over time. This contrast gives rise to variations in the fossil record within limestone and helps scientists better understand the organisms that contributed to its formation [49]. Moreover, he was the first to observe calcitisation—what he referred to as a “molecular change”. The value of Sorby’s pioneering applications of microscopy to study the layered structure of nacre has been immense, with significant repercussions for modern directions in biomineralisation and materials science. Methods and findings have formed the basis for continued study into the origin and properties not only of biominerals but also the creation of new biomimetic materials. Sorby found that aragonitic fossils do not behave like calcitic ones during diagenesis. Where calcitic fossils still retain their original shell structure, aragonitic fossils completely lose this integrity. Aragonite is a less stable form of calcium carbonate and often recrystallises to calcite or completely dissolves during diagenesis, which destroys the original aragonitic shell structure [50,51,52].

### 2.4. 1900s: Understanding Nacre’s Structure

#### 2.4.1. Early–Mid-1900s (1900–1960)

There was significant development related to the chemical and mechanical characterisation of nacre during the early-to-mid-1900s. In 1917, Clarke and Wheeler [53] contributed considerably to the knowledge about the chemical composition of different types of shells. Their investigation showed that the major constituents of nacre include calcium carbonate, usually as aragonite, together with a small amount of organic matrix matter. This organic matrix serves as the binder and is crucial to the structure, formation, and mechanical properties of nacre. Their work showed how inorganic calcium carbonate interacted with organic material to produce a distinct material, namely nacre, characterised by its properties and functional features.

In 1930, Bøggild published his book *The Shell Structure of Mollusks* [54], in which he distinguished between the mineralogical structures of aragonite and calcite in mollusc shells. His study of the nacre layer revealed the fundamental differences in the crystalline arrangement and properties of these two common biominerals found in the shells of many marine organisms.

In 1942, R. Trueman investigated the structure and growth of the shell in the species *Tellina tenuis*. His work detailed the mantle structure and the distribution of calcium within it, thus adding to earlier studies that described the structure. All these factors further enhanced the broader understanding of the shell’s structural details. Traces of strontium were present in *T. tenuis*, and magnesium, present in many calcite shells and other aragonite shells, indicates the participation of some non-calcareous substances in the calcite or aragonite structures. Lastly, from an environmental perspective, this research examined the effects of temperature and salinity, which may significantly influence shell development, thereby enhancing our understanding of this dynamism. This multidimensional approach provides a perspective on the nature of shell formation in bivalve molluscs, encompassing structural analysis, physiology, and environmental factors. Knowledge is advanced, with regard to *T. tenuis*, towards the knowledge base of the wider disciplines of malacology and biomineralisation [55].

In 1956, Francis G. Stehli published a study in *Science* on the shell mineralogy of Palaeozoic invertebrate groups. In his research, Stehli concluded that recrystallised shells or shell layers are highly reliable indicators of a primary aragonitic shell mineralogy. This discovery reveals the complex structural and compositional features of these ancient marine organisms, thereby enabling a deeper understanding of the evolutionary adaptations and environmental influences that have shaped their development [56]. In the next year (1957), C. Grégoire [57] and K. Wada in 1961 [58] used SEM to study nacre materials; they confirmed the presence of tabular aragonite crystals arranged in the fashion of “brick-wall” construction.

XRD has become an indispensable tool for characterising the structures of both inorganic and organic materials. It provided detailed information on crystal structures, phases, and other structural parameters [59,60,61]. The technique was used to study the arrangement of aragonite platelets in nacre (mother of pearl), a classic model in biomineralisation. These latter studies showed the coexistence of flat and curved platelets and a gradient in their morphology and thickness, which was interpreted as resulting from differences in growth rates and compressive stress [62,63]. The application of XRD in the early-to-mid-1900s significantly advanced the understanding of material structures at the atomic level. This period saw the development of methods to analyse complex structures, including biological macromolecules and synthetic materials [64,65]. XRD patterns of nacre typically show characteristic peaks for aragonite, with the main reflection at around 31.05° 2θ and two lesser reflections detected at 33.0° 2θ and 66.0° 2θ [66]. The XRD analysis of nacre in mussel shells demonstrated that, in nacre, the (002) crystal plane of aragonite is strongly preferentially oriented (interplanar spacing d = 0.288332 nm), with other reflections such as the (014) and (012) planes appearing relatively weakly represented [67]. This preferred orientation is a hallmark of the highly organised structure of the platelets of aragonite within nacre.

#### 2.4.2. Late 1900s (1960–2000s)

The late 1900s (1960–1990s) saw significant advancements in understanding the mechanical properties of nacre, particularly its strength and toughness. Early theories on crack propagation and energy dissipation mechanisms, such as platelet sliding, crack bridging, and hierarchical toughening, have made an important contribution in explaining nacre’s remarkable mechanical performance.

Wada [58] demonstrates that shell formation is highly complex and significantly influenced by both biological mechanisms and environmental conditions. The interaction between the metabolic processes of the mollusc and the chemistry of seawater is essential in determining the characteristics of the shell. The organic matrix, composed of various proteins and polysaccharides, plays a pivotal role in crystal nucleation and growth, thereby underscoring the importance of biological materials in mineralised structures, a key finding of the recent study. Furthermore, molluscs exhibit adaptability in their shell growth in response to fluctuating environmental conditions such as temperature, pH, and salinity. This adaptability is significant for understanding how these organisms may cope with changing ocean environments, particularly in the context of climate change.

Dr John D. Taylor is a Scientific Associate in the Department of Life Sciences at the Natural History Museum, London. His research focuses on the biology, systematics, and evolution of chemosymbiotic bivalve molluscs. His book makes a significant contribution to understanding the literature on nacre and bivalve molluscs, particularly their shell structure and mineral composition. It provides a detailed examination of bivalve shell structures, focusing on the families Nuculacea and Trigonacea, and offers thorough descriptions of the layers and materials that form the shells, with attention to their biological functions and evolutionary adaptations. The book also explores the mineralogy of bivalve shells, identifying the types of minerals present and their importance for reconstructing past environments and understanding ecological roles. Through a comparative analysis of shell structures and mineral compositions across different bivalve groups, the study identifies evolutionary trends and adaptations, enhancing knowledge in evolutionary biology. Additionally, it establishes a framework for interpreting fossilised bivalve remains, offering valuable insights for palaeontologists studying the history of marine life [68].

In 1968, Towe and Hamilton published a paper titled “Ultrastructure and inferred calcification of the mature and developing nacre in bivalve mollusks” [69], which represents a significant contribution to our understanding of the structure and formation of nacre in bivalve molluscs. It provides a detailed analysis of the nacreous layers in certain species, confirming the presence of tabular aragonite polygons interspersed with a perforated organic matrix. In their research, the authors have challenged prevailing views on the nature of tabular aragonite crystals, arguing that these are composed of smaller component blocks rather than single crystals. The study highlights the important role of the organic matrix in nacre formation, which is secreted by mantle tissues before calcification begins. Based on this, the authors propose a hypothesis regarding the mechanism of nacre formation and further develop a dynamic interpretation of shell formation based on available evidence. Furthermore, high-resolution studies of the structure of nacre using electron microscopy give good confirmation that immature nacre has hollow “crystals” and thin “crusts”, which are highly important to understanding how mature nacre is formed. These studies, in turn, make a major contribution to the realisation of the remarkable properties of nacre and the complex biological processes that form mollusc shells.

In 1969, Gerrit Bevelander and Hiroshi Nakahara [70], of the Department of Histology at The University of Texas Dental Branch and The Bermuda Biological Station for Research, used electron microscopy to study the structural relationships between the pearl sac epithelium, nacreous layers, and surrounding fluid, expanding our knowledge of nacre formation. Specimens of bivalve molluscs, such as *Pinctada radiata*, *Mytilus exustus*, and *Anomia simplex*, were collected and chilled at 5 °C for one hour to anaesthetise the tissue, minimising shrinkage during fixation. The adductor muscle was cut into halves and immersed in cold fixatives consisting of 1% osmium tetroxide in phosphate buffer, pH 7.4, for 1–2 h and then 6% glutaraldehyde in phosphate buffer, pH 7.4, for 6 h, with post-fixation in osmium tetroxide for an additional hour. These specimens were further dehydrated, embedded in Araldite, and cut into thin sections to study the inner shell surface in contact with the mantle epithelium. Selected sections were stained on 2% lead citrate, visualised on the electron microscope, and prepared as replicas of the inner shell surface using a single-step collodion technique. Similarly, fixed, embedded pearls along with their sacs were prepared for microscopy. Their study represents a valuable addition to the literature on nacre formation. It reveals the initial step, where the polymerisation of the pallial fluid forms lamellae parallel to the epithelial surface. In addition, the pallial fluid controls crystal growth and arrangement, providing the organic components necessary for nacre. Moreover, the compartments that develop during growth ensure a consistent crystal thickness and orientation, contributing to both structural integrity and the appearance of nacre. Such a study points to an induction mechanism in which the organic matrix influences the nucleation of new crystals near and around existing crystals. These studies lay the foundation for how nacre layers are deposited uniformly and how the lamellar thickness is kept constant during the course of their growth [66,70,71].

In 1969, Iwao Kobayashi [72] described the structure of bivalve shells using both light and electron microscopy. Light microscopy showed general morphology, while electron microscopy furnished detailed information about the submicrostructure. The calcareous shell layers are divided into eleven (11) different basic types based on the morphological features observed under the microscope. The following describes in detail the morphological types of calcium carbonate structures observable under a microscope that are commonly found in biological materials, such as shells and skeletal structures. The structures are differentiated based on their basic units, morphological features, and associated organic phases. The prismatic type consists of polygonal prisms with rods running parallel in one or more directions, composed of rectangular rods, encircled by an eosinophilic sheath and a basophilic membrane that contains collagen-like fibres or amorphous matter. The fibrous prismatic type is also composed of one-directionally aligned rectangular rods, associated with basophilic fibrous or membranous matter forming a lace-like reticulated sheet. The nacreous type consists of narrow tablets aggregating into broad tablets, enveloped by a basophilic sheet and fibrous matter. The foliated type consists of elongate tablets aligned in one or more directions, giving a resemblance to cross-lamination, and there is a basophilic sheet. The features and organic phases of the trans-prismatic type, probably composed of very fine rods, are unknown. The crossed lamellar type consists of rectangular rods that form first-order lamellae, with rods aligned in one direction and adjacent lamellae with rods inclined in opposite directions. Basophilic fibres and an eosinophilic matrix are associated with the rods. The pseudo-crossed lamellar type differs in having much smaller, irregular first-order lamellae and is of unknown organic phases. The complex type consists of rectangular rods aggregated into blocks, associated with basophilic fibres. The composite prismatic type consists of rods resembling pine branches that form prisms surrounded by basophilic fibrous matter and an eosinophilic matrix. The fibrous type consists of rectangular rods roughly aligned in the same direction, with basophilic fibres present. Finally, the homogeneous type has a granular structure with irregular or regular aggregation associated with basophilic, partly eosinophilic matter. These structures typically exhibit configurations of basic units and organic phases that can be identified by staining microscopy, showing eosinophilic (acidic) and basophilic (basic) components, which may inform our understanding of their formation and classification.

Advances in microscope (SEM, TEM) allowed researchers to study nacre’s hierarchical structure in detail. Taylor, Kennedy, and Hall published their second book in 1973, titled *The Shell Structure and Mineralogy of the Bivalvia. II. Lucinacea—Clavagellacea Conclusions* [73]. This publication focuses on the microstructure and composition of bivalve shells. It examines the shell characteristics of various bivalve superfamilies, with an emphasis on their mineralogy and structural layers. The book provides descriptions of shell structures, including crossed-lamellar, complex crossed-lamellar, and composite prismatic layers, alongside their arrangements across different bivalve species. This work is a significant reference for the study of bivalve shell evolution, classification, and adaptation to diverse environments [68,73,74]. Currey’s 1977 work quantified the mechanical properties of nacre, revealing its high toughness and fracture resistance [75]. This laid the foundation for studies on the macroscopic mechanical properties of nacre.

In 1979, Stephen Weiner [76] used ion-exchange chromatography to isolate organic components from various molluscs. This enabled the identification of aspartic acid-rich proteins, which are integral components of the soluble matrix. The research demonstrates the potential for comparative studies across different mollusc classes, given the wide representation of molluscs examined. Furthermore, it enhances our understanding of the biochemical roles of these proteins in mineralisation. Weiner concludes that the presence of these proteins in both mollusc shells and vertebrate dentine demonstrates their fundamental importance in mineralisation processes across diverse biological systems, indicating an evolutionary conservation of these proteins.

In 1992, Mehmet Sarikaya and Ilhan A. Aksay authored a chapter entitled “Nacre of Abalone Shell: a Natural Multifunctional Nanolaminated Ceramic-Polymer Composite Material” [77] in the book titled *Structure, Cellular Synthesis and Assembly of Biopolymers* [78]. At that time, the authors concluded that, although the detailed mechanisms underlying such toughening and strengthening are not yet well understood, it is becoming clear that the hierarchical organisation of aragonite platelets, twin-defect structures within them, and their interactions with the organic matrix may play a key role. Mechanical properties, the composition and role of the organic matrix, growth mechanisms, and bio-duplication were critical areas requiring further study. The knowledge gaps identified were:

(a) **Mechanical Properties:** Determine the toughening and strengthening mechanisms due to organic–inorganic interactions; shell property evaluations under dynamic conditions shall inform biomimetic material design.
(b) **Organic Matrix:** Establish the composition of proteins, their conformation, their interaction with aragonite, and their correlation to calcite at the prismatic layer to inform material design.
(c) **Growth Mechanisms:** Illustrate how inorganic nucleation and growth occur at each developmental stage under the influence of the organic matrix, thus illuminating the organic control of shell development and size.
(d) **Bioduplication:** Study self-assemblies that isolate, purify, and reassemble organic matrix components under the controlled mineralisation and synthesis of hard biological structures.

The authors of this chapter mentioned this statement: “Designing and processing novel materials similar to the multifunctional, nanolaminated composites inspired by nacre through biomimicking approaches, and eventually through bioduplication, will have to wait until we answer the crucial questions regarding the structure and function of this system.” Their research framework at that time aimed to develop nacre-inspired approaches to advanced nanomaterials, with a focus on biomimetic design rules and controlled synthesis techniques for multifunctional materials.

In 1997, Tilman E. Schäffer et al. [79] examined the growth of abalone nacre, focusing on the influence of mineral bridges and the architecture of interlamellar organic sheets. Atomic Force Microscopy (AFM), Scanning Ion Conductance Microscopy (SICM), scanning electron microscopy (SEM), and transmission electron microscopy (TEM) were used to analyse the structural and morphological properties of nacre. The samples were prepared for AFM imaging through the “flat pearl” on the cover glass by a demineralisation of the samples in order to obtain clear images of surface features. Other experiments to characterise the organic matrix thickness and pore structure employed SICM. For these tests, the specimens were rinsed and then sealed in a chamber containing a KCl solution for scanning with a micropipette. Scanning electron microscopy, after being rinsed and dried, gave the surface morphologies of the nacre tablets. Using TEM, high-resolution imaging of its internal structure was achieved by preparing thin sections from fixed, resin-embedded nacre samples. Other functional analyses, such as demineralisation with acetic acid and enzymatic digestion with proteinase K and collagenase, were also used to isolate and analyse the organic components. The study concluded that abalone nacre grows by mineral bridge formation through interlamellar organic sheets rather than heteroepitaxial nucleation, and this conclusion is supported by extensive experimental observations. These interlamellar sheets were found to contain pores 5–50 nm in diameter, through which the traffic of ions and soluble proteins occurs to affect aragonite crystal growth. During aragonite tablet growth, they initially grow vertically until they reach the next interlamellar sheet, after which growth shifts laterally through the pore outwards, thereby ensuring a coherent alignment of crystal axes across successive tablets. Transmission and scanning electron microscopy revealed mineral bridges across the tablets, which are important for maintaining structural integrity and supporting nacre growth. Quantitative analyses further showed that pore-to-pore spacing correlates with the lateral offsets of the nacreous tablets, supporting the conclusion that crystal growth is influenced by features of the organic matrix structure. These findings represent an important step in advancing the knowledge of biofabrication in gastropod nacre, emphasising the crucial role of mineral bridges and specific organic sheet properties in enabling nacre formation.

### 2.5. Rise of Biomimicry and Controlled Fabrication

At the beginning of the second millennium and in recent decades, nacre has inspired researchers to develop various artificial materials due to its exceptional mechanical properties. Its complex structure has been replicated; synthetic versions are now being engineered for advanced materials used in engineering and biomedicine for orthopaedic and soft-tissue applications, thereby demonstrating its continued relevance and suitability for emerging needs [80,81,82,83].

In the early 2000s, Chateigner, Hedegaard, and Wenk [84] studied mollusc shells by combining X-ray diffraction with scanning electron microscopy. They used XRD to characterise the orientation distribution of crystallites within the aragonite layers and SEM to obtain high-resolution images of shell microstructures, thereby linking the two data sets at high resolution. The study showed that crystallographic textures across mollusc groups, even within those with similar microstructures, varied substantially, with no straightforward one-to-one correspondence between microstructure and crystallographic orientation. This was evident in crossed-lamellar layers, which appeared morphologically similar but exhibited different crystallographic textures across species, with variations in texture strength occurring within layers of a single species. This indicates that crystallographic textures and microstructures provide independent, complementary phylogenetic indicators and enable a deeper understanding of evolutionary relationships among molluscs.

In 2003, the paper entitled “Nanostructured Artificial Nacre” by Tang et al. [85] was a significant milestone in materials science regarding biomimetic composite development. The authors reported the creation of an artificial nacre-inspired material composed of successive layers of polyelectrolyte and clay that exhibited improved mechanical properties due to stress-hardening effects. They also showed how ionic interactions determine mechanical strength and how ion exchange influences properties by disrupting ionic bonds, and further discussed the conformational states of the polyelectrolyte molecules in the composite. Moreover, the authors demonstrated that the Young’s modulus of their artificial nacre is at least as high as in natural materials and presented their work as a breakthrough in biomimetic materials, which would be highly influential for the design of new tough and resilient materials.

In 2006, Sylvain Deville et al. [86], for the first time, proposed freezing as a novel technique to prepare complex composite materials using the physics of ice formation in their paper “Freezing as a Path to Build Complex Composites”. Freezing enhances mechanical properties by promoting microstructural homogeneity, improving interfacial bonding, and reducing voids and cracks, thereby enhancing strength and toughness. The control of the freezing rate yields properties ranging from rigidity to flexibility, while improved thermal stability extends the range of applications to extreme conditions. They demonstrated that their technique could be applied across multidisciplinary fields, including construction, aerospace, and the biomedical industry, where performance is governed by the intricate structures of materials. This process is less energy-intensive, thereby meeting sustainability objectives and enabling the adoption of greener technologies.

Munch, E. et al. [87] applied freeze casting in 2008 to fabricate large porous ceramic scaffolds by carefully controlling the freezing of ceramic-based suspensions in water. In this process, ice crystals act as templates for the formation of layered structures that are subsequently infiltrated with a polymer phase. This approach enables the making of lamellar ice with specified dimensions as a negative mould for fabricating layered ceramic scaffolds. The method enables a controlled architecture in the resulting materials. After the ceramic scaffolds were fabricated, a sintering treatment was applied to densify the structures and to facilitate the formation of ceramic bridges among the layers. It is one of the steps necessary to enhance the mechanical properties of the products. In the work reported here, chemical grafting of the ceramic surfaces was also performed before polymer infiltration. Grafting methacrylate groups onto ceramic surfaces is expected to enhance the adhesion between inorganic and organic phases, thereby increasing the strength and toughness of such hybrid materials. Nonlinear elastic fracture mechanics, particularly the J-R curve approach, have been used to evaluate the material’s toughness. The results obtained using this method enabled the characterisation of toughness during crack growth, thereby providing a greater understanding of the mechanical behaviour of these materials under stress. Moreover, in situ fracture toughness tests were conducted in a scanning electron microscope to investigate damage mechanisms and toughening processes in real time. Such tests provided further insight into how materials respond to loading and how the toughening mechanisms operate at different scales.

#### 2.5.1. Nacre-Inspired 3D Printing

The unique brick-and-mortar nacre structure has been successfully demonstrated for 3D printing in various composite materials, with several studies demonstrating its potential and advantages [88,89]. Various fabrication techniques, including fused deposition modelling (FDM), have been employed to develop nacre-inspired structures with enhanced mechanical properties. Additionally, a newly developed direct-write 4D-printing approach has been utilised to fabricate smart, nacre-inspired organic-ceramic composites comprising alumina platelets and vitrimers, achieving tensile strengths and stiffnesses up to 3.3 and 26.7 times higher, respectively. The approach will enable the creation of smart composites capable of self-healing and exhibiting shape memory. Moreover, digital light processing (DLP) has successfully aligned platelets within the ultraviolet resin, thereby enhancing thermal conductivity [90].

In 2013, Dimas et al. [91] 3D-printed nacre-inspired structures with precise architectural control, employing mesoscale molecular mechanics models to analyse these composite structures. These computational techniques have been instrumental in simulating bioinspired composite behaviours, identifying optimal topological arrangements for toughness and crack-propagation stability, and predicting responses under various mechanical loads. The experimental results were compared with computational predictions to confirm that the synthesised composites exhibited improved mechanical properties, including the stiffness, maximum strain, maximum stress, and toughness modulus, relative to the individual components. This work demonstrated the potential of computational modelling for designing composite materials with prescribed fracture properties that can be fabricated using advanced 3D printing techniques and underlined the strong integration of computational design and additive manufacturing in materials science [92,93,94,95]. More recently, additive manufacturing, or 3D printing, has been employed to replicate the structure of nacre by manufacturing composite materials composed of stiff and soft polymers in an alternating layered geometry. The process provides a good control over geometry and material properties, enabling the manufacture of structures with mechanical performance comparable to that of natural nacre [96,97].

**Figure 3:**
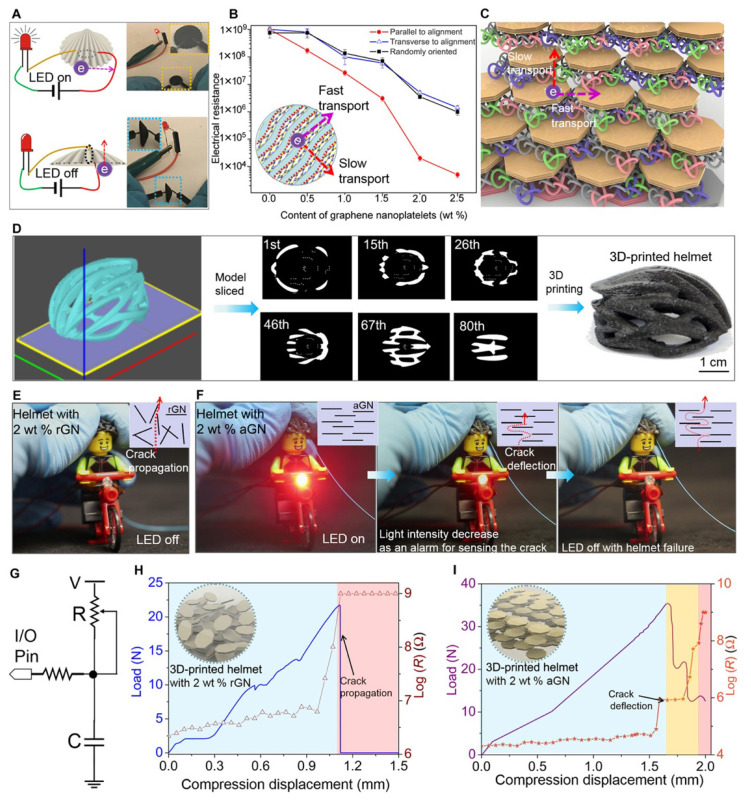
3D-printed smart helmet with anisotropic electrical property. (**A**) Anisotropic electrical property of the 3D-printed nacre. (**B**) Changes in electrical resistance with different GN loadings and alignments. (**C**) Schematic diagram showing the layered polymer/GN structure with anisotropic electrical resistance. (**D**) 3D printing process of a self-sensing smart helmet. Demonstration of the wearable sensor on a Lego bicycle rider showing different self-sensing properties for the 3D-printed helmets with rGNs (**E**) and aGNs (**F**). (**G**) Circuit design for the tests. Compression force of the 3D-printed helmets with related compression displacements and resistance changes for rGNs (**H**) and aGNs (**I**), respectively. (Photo credit: Yang Yang, Epstein Department of Industrial and Systems Engineering, University of Southern California). Reproduced with permission from *Science Advances* [98].

In 2019, Yang Yang et al. [98] fabricated nacre-inspired hierarchical structures with complicated three-dimensional (3D) shapes by means of electrically assisted 3D printing, as shown in Figure 3. They incorporated graphene nanoplatelets (2 weight %) into the polymer matrix during the 3D printing process, resulting in a lightweight material (1.06 g/cm^3^) that exhibits a specific toughness and strength comparable to natural nacre. This is significant for applications involving lightweight yet strong materials, such as military and sport armours. The novelty of this work lies in the development of self-sensing features within the 3D-printed structure. Integrated, aligned GNs enable the material to detect various forms of damage via changes in electrical resistance, an essential feature for smart materials used in personal protective gear. Yang Yang et al. showed that the electrically assisted 3D printing process can yield smart structures that are not only lightweight and strong but also capable of sensing their own integrity. This opens new avenues for use in biomedical engineering, space, and the military.

In 2022, Yang Yang et al. [99] showed a compelling and innovative approach to materials design, achieving a remarkable synergy of mechanical, thermal, and flame-retardant properties through advanced 3D printing and bioinspired material design. The demonstrated enhancements over natural nacre are substantial and open significant avenues for both fundamental research in materials science and real-world applications. The integration of amorphous boron nitride (a-BNs) into a novel grafting technique for 3D printing enables a versatile platform for creating high-performance materials. This material represents a significant advance in protective material design, particularly given the reported substantial increase in thermal protection performance. Moreover, the nacre-inspired structure exhibits outstanding mechanical and fire-resistant properties and was fabricated via a rotation-blade casting 3D printing method. In this process, 3-propyl methacrylate-coated boron nitride nanoplatelets (BNs) are aligned through rotation-blade casting to build up the “brick and mortar” architecture during the 3D printing process [99].

#### 2.5.2. Nacre-Inspired Sustainable Material

**Figure 4:**
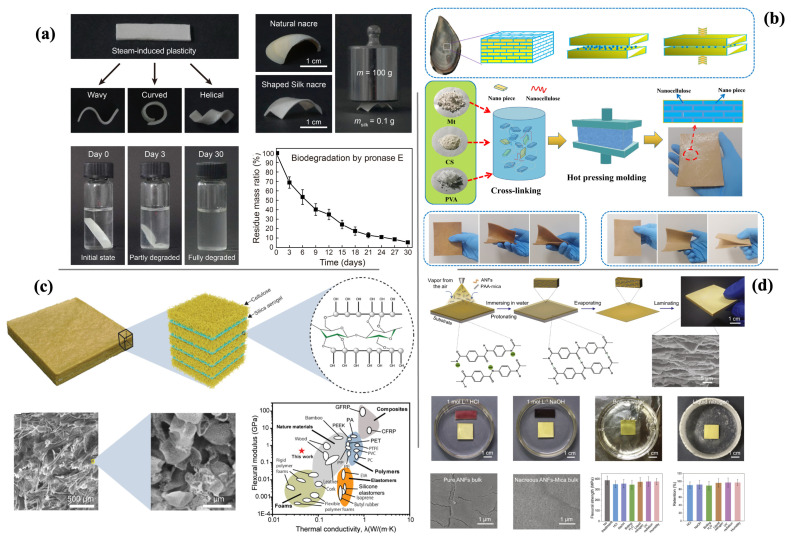
Examples of nacre-inspired sustainable material (**a**) plasticity and biodegradability of the silk nacre, reproduced with permission from The American Association for the Advancement of Science [110]. (**b**) Multiscale interface design and fabrication of nanocomposites, reproduced with permission from Springer Nature [109]. (**c**) Predried nanoporous silica/cellulose layered composite, adapted with permission from [107]. Copyright 2024 American Chemical Society. (**d**) Fabrication of the nacreous ANF-Mica bulk and its environment, reproduced with permission from Elsevier [114].

The inspiration for sustainable materials derived from nacre is characterised by strength, toughness, and light weight, with a layered or lamellar structure [100,101,102]. These materials combine a high strength and high fracture toughness and are therefore suitable for producing alternative, sustainable, high-performance structural and functional materials. Nacre has a very unique “brick-and-mortar” structure composed of 95 vol% of brittle inorganic minerals and 5% organic polymers, which is responsible for its extraordinary mechanical properties, including an ultra-high work of fracture and excellent bending strength [101,103]. Nacre-inspired sustainable materials contribute to environmental conservation by potentially substituting for conventionally used synthetic materials that have significant environmental impacts, including steel and plastics [104]. These materials are promising solutions for developing advanced, sustainable materials in response to evolving environmental requirements, including energy-absorbing lightweight structures for various impact applications [105]. Nacre-inspired sustainable materials have become one of the points of focus for researchers trying to replicate the astonishing properties of natural nacre in man-made material systems, as shown in Figure 4. Among these, cellulose-based composites show strength and thermal stability in addition to their environmental friendliness. TEMPO-oxidised wood, which is usually produced from a composite of TEMPO-oxidised wood and polyvinyl alcohol, upon reinforcement with borax, creates a high tensile and flexural strength, thermal stability, and flame retardancy [106]. In addition, cellulose–silica aerogels, which merge the structural strengths of cellulose and the thermal insulation of silica, produce materials featuring excellent mechanical properties with low thermal conductivity [107]. In the field of biodegradable composites, chitosan-based materials represent an environmentally friendly alternative to conventional plastics, inspired by the hierarchical structure of nacre and amenable to large-scale production [108]. Montmorillonite/chitosan/PVA composites mimic some of the structural features of nacre and show very high flexural strength, fracture toughness, and fire resistance properties, ideal for engineering applications [109]. Advanced fabrication techniques have enabled the development of high-performance structural materials with superior mechanical and thermal properties. To mention a few, the directional deforming assembly method was utilised to produce cellulose nanofibers/mica microplatelets with enhanced strength, toughness, and thermal stability [104]. Additionally, silk nacre is a single-component composite composed of silk fibroin; the self-similarity in its layered structure renders it nacre-like and confers mechanical properties superior to those of natural nacre, with enhanced biodegradability [110]. Possible localised energy absorption and interlayered delamination in the hierarchical structure of nacre-inspired materials allow for the great enhancement of the weight-saving and ballistic performance of protective structures [105]. Some of the challenges that must be overcome in developing nacre-inspired sustainable materials for industrial applications include the complex processing, high energy consumption, and limited scalability of existing preparation techniques [103]. Other challenges involve refining nacre-inspired architecture and fabricating large-scale materials under controlled conditions [111]. In addition, it is necessary to increase the fraction of inorganic components in artificial materials with an ordered inner brick-and-mortar structure up to 95 vol% and to enhance the interface effect in artificial materials [111]. Future developments in research on nacre-inspired sustainable materials include the synthesis of new materials with improved mechanical properties and stability, such as high-strength integrated artificial nacre based on sodium carboxymethylcellulose and borate cross-linked graphene oxide sheets [112]. The development of high-strength integrated artificial nacre through the sequential infiltration of polymer-stabilised CaCO_3_ liquid precursors into layers of pre-deposited nanocellulose films shows the potential to create fully sustainable composite materials with outstanding properties using abundant biopolymers and biominerals [113].

#### 2.5.3. Wooden Artificial Nacres (WANs)

**Figure 5:**
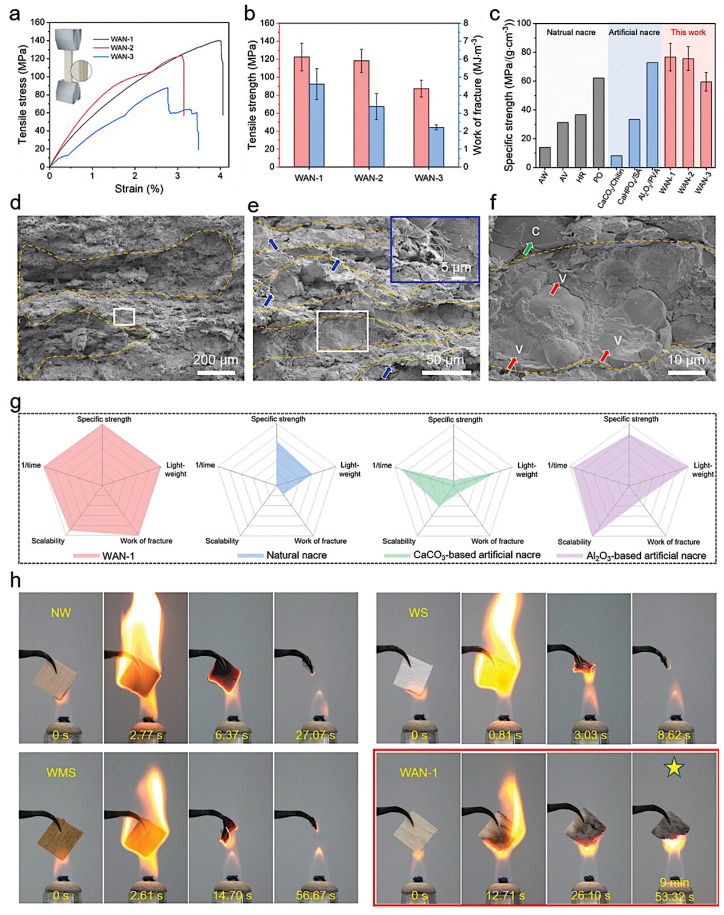
Tensile properties of WANs. (**a**) Tensile stress–strain curves of WAN. (**b**) Strength and work of fracture of WANs. (**c**) Comparison of the specific strength of WANs, natural nacre (AV: Atrina vexillum), and bulk organo-inorganic artificial composite nacre with different inorganic materials. (**d**) SEM image of the fracture section of WAN-1; the yellow areas are the holes which were created by tensile fracture of pull-out. (**e**) The “brick-and-mortar” structure (yellow dot lines) and pulling-out micro-fibrils in WMS parts (blue arrows). (**f**) SEM image of fractured vaterite (red arrows) and calcite (green arrow). (**g**) Comparison of radar maps of WNA-1 with other organic–inorganic artificial composites in terms of specific strength, light weight, work of fracture, scalability, and time efficiency. (**h**) Flame combustion experiments of NW, WS, WMS, and WAN-1. The photographs in each group depict the initial stage, the maximum-flame stage, the flame-extinguishing stage, and the end of burning (from left to right). Reproduced with permission from John Wiley and Sons [103].

The design of wooden artificial nacres (WANs) is intended to effectively mimic the natural structure of nacre by combining the advantages of wood with most of nacre’s structural features, yielding composites with outstanding mechanical properties, as shown in Figure 5. WANs have a hierarchical, layered structure similar to natural nacre, incorporating multi-hierarchical lamellar arrangements, organic bridges, and micro-asperities [103,115], achieved through processes such as mechanical/chemical mineralisation and assembly [116]. Lignocellulose, a lightweight and abundant material showing an outstanding mechanical performance, is the main feedstock of WANs [116]. In the study conducted by Z. Qiu et al. [103], WANs exhibit excellent mechanical properties, such as a bending strength of about 93.31 MPa, toughness around 7.40 MPa m^1/2^, tensile strength of about 122.59 MPa, and work of fracture of about 4.61 MJ m^−3^, while maintaining a low density of about 1.59 g cm^−3^, which leads to excellent specific mechanical properties compared to both natural nacre and other artificial nacre materials. These advantages of WANs include achieving an almost equivalent strength to natural nacre with a significantly reduced inorganic content, enhanced scalability in production processes that overcome the limitations of traditional nacre-inspired materials, and sustainability by using wood as a renewable resource, thus providing an eco-friendly alternative for engineering applications [103,117]. With a high specific strength, toughness, and dimensional stability, WANs serve as a building block for sustainable, biomimetic lightweight materials with superior mechanical properties and broad prospects for global high-performance organic–inorganic composite engineering [118].

#### 2.5.4. Machine Learning

In 2018, Grace X. Gu et al. [119] developed a new approach that integrates machine learning with finite element analysis for advanced materials design. It uses a convolutional neural network with a self-learning algorithm to optimise material properties based on patterns discovered in a large database of FEM-derived geometries. One of the major innovations in this paper is the application of machine learning to coarse-graining, condensing numerous building blocks into a single unit cell, thereby reducing the model complexity. A simple material model is also adopted to balance computational cost with a single stiff and a single soft component. The validity of the designs proposed by the ML model is further verified through 3D printing and tensile tests. The approach identifies high-performing designs by identifying microstructural patterns that enhance material toughness and strength, underscoring the critical role of unit-cell optimisation in maximising the properties of composite materials.

**Figure 6:**
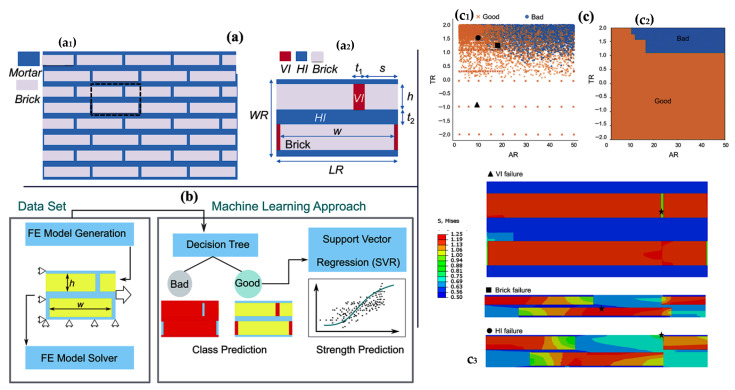
(**a**) The brick and mortar architecture and the representative volume element. (**a_1_**) brick-and-mortar composite microstructure. Bricks are arranged in a staggered pattern, which are perfectly bonded with the mortar. The mortar (soft and ductile) and the bricks (hard and brittle) are represented by blue and grey, respectively. (**a_2_**) The selected RVE (representative volume element) with the geometrical features. The mortar is divided into two sections: the vertical and horizontal sections, which for clarity are coloured red and blue, respectively. symbol *VI* and *HI* are the vertical and horizontal interfaces, respectively. *w* and *h* are the brick length and height, respectively. t_1_ and t_2_ are mortar vertical and horizontal thickness, respectively, and *s* is the shift in stagger arrangement; (**b**) data generation and ML model development procedure, the data set was generated by MATLAB and ABAQUS. Mechanical strengths obtained from FEA were considered as the ground truth and held the correct class. The classification to good and bad classes was done through the decision tree with its leaves representing different features conditions. A support vector regression was used for strength prediction; (**c**) boundaries of the good and bad design in design space and the stress distribution in selected points, (**c_1_**) Scatterplot of good and bad designs in TR-AR design space for D training. The response is colour coded: the orange cross and blue circle represent points in the good and bad classes, respectively. (**c_2_**) The partitioned space by the decision tree. Orange and blue correspond to good and bad classes, respectively. (**c_3_**) The von Mises stress distribution obtained from FEA for selected points shown in a. The stress contours are von Misses stress, and the figures are undeformed geometry of the RVE. The stress magnitude is normalised with respect to the applied load. The selected points illustrate the different failure possibilities: the black triangle represents a sample in the good class (*VI* fails first). The black circle and square are examples of bad designs with *HI* and brick failure, respectively. The red colour shows the region with the highest stress value. All the geometries in c have the same value of *s* = 0.2 and *v_m_* = 0.5 while they present different failure modes. The heights of RVE are 0.43, 0.1, and 0.15 for the points shown by the triangle, circle, and square point in a, respectively. The stars in (**c**) show the location of the failure initiation. Failure is considered as the onset of yielding in the mortar and fracture of the ceramics. TR (horizontal mortar ratio), AR (the aspect ratio of the brick), *v_m_* mortar volume fraction, and *s* (shifting distance of the bricks) [120].

As shown in Figure 6, in 2020, Seyedreza Morsali et al. [120] reported a new computational approach that integrates statistical analysis, machine learning, and finite element analysis for the design of strong brick-and-mortar composites inspired by nacre. They probed more than 20,000 geometries using systematic and random sampling; therefore, the current study was able to comprehensively scan a wide design space. Focusing on independent geometrical features, such as the aspect ratio (AR), thickness ratio (TR), and mortar volume fraction ($v_{m}$), the geometries were classified as “good” and “bad” designs based on the failure modes. Good designs were identified as those that could carry loads without causing the vertical mortar to fail. The statistical analysis showed that the aspect ratio of bricks and horizontal mortar thickness were the most influential factors on the design performance. Systematic sampling was supplemented with random sampling within predefined feature ranges to obtain a broad representation of the design space. The results were visualised as box plots, which showed that the distributions of AR and TR values for good and bad designs were completely separable. It introduces a new computational framework that integrates statistical analysis, machine learning (ML), and finite element analysis to develop effective structure–property design strategies for brick-and-mortar composites, thereby enabling the efficient exploration of very large design spaces. Some machine learning models were developed using decision trees and support vector regression (SVR) to predict strength and rank geometries, thereby demonstrating the potential of ML in optimisation for design selection. Several parameters governed composite strength, such as TR and $v_{m}$, and provided greater detail on the design trade-offs.

#### 2.5.5. Nacre-Inspired Graphene-Based Materials

Graphene-based nacre-inspired structures have been one of the most critical research areas due to the outstanding mechanical properties of graphene. Graphene-based nacre-inspired structures, which mimic the brick-and-mortar structure of natural nacre, exhibit a significantly higher tensile strength, toughness, and energy absorption than natural nacre. The main forces that maintain the structural integrity include van der Waals forces, hydrogen bonding, ionic coordination, and covalent crosslinking [121,122,123,124]. These materials have a wide range of applications, ranging from flexible electronics that require mechanical and electrical conductivity [125,126] to the aerospace industry for their light weight and strength properties. Their resilience and flexibility also enable applications in artificial muscles and tissue engineering [112,125,127]. A newly reported “Photothermal Energy Cascade” approach based on colloidal graphene and thermoreversible supramolecular bonding enables an adaptive variation in stiffness and toughness under light stimuli [128].

#### 2.5.6. Nacre-Inspired Smart Materials

Nacre-inspired smart materials represent one of the most fascinating fields in which unique features in nacre structure are combined with advanced functionalities typical of smart materials. These materials are designed by biomimicking the nacre brick-and-mortar structure, which exhibits outstanding mechanical properties such as strength and toughness, yet is lightweight [128,129,130]. In addition, the unique architecture of nacre-inspired structural materials confers excellent mechanical properties, outperforming those of their constituent phases and making them superior to traditional smart materials [100]. These nacre-inspired smart materials include:

##### Nacre-Inspired Self-Healing Materials

Nacre-inspired self-healing materials exploit nanoparticle migration to damaged regions to facilitate repair, mimicking natural healing processes. Nacre-inspired self-healing materials exhibit autonomous, site-specific healing of damage, resembling the dynamic nature of natural living systems [131]. These materials exhibit impressive self-healing performance, and some can heal even at room temperature. The PU-graphene oxide network showed an 88.6% self-healing efficiency at 25 °C after 24 h. Moreover, some designs can achieve rapid self-healing [132]. A biomimetic nanocomposite coating with dynamic supramolecular hydrogen bonds can self-heal efficiently within just 10 min at room temperature [27]. The hierarchical structure, dynamic bonding, strong interfacial interactions, microcapsules containing healing agents, and nanoparticle migration are key factors in the microstructure of nacre-inspired self-healing materials that enable self-repair.

(a) Hierarchical Structure: Nacre-inspired materials often mimic the hierarchical micro/nanosized structure of natural nacre by using a combination of alternating layers of hard and soft materials. Such a unique hierarchy can provide a balanced stiffness, toughness, and flexibility. At the top of such a hierarchy, there is a nanosized mineral bridge between neighbouring platelets that connects them, enormously decreasing the level of stress and preventing organic polymer matrix failure [133,134].
(b) Dynamic Bonding: These materials include dynamic bonds such as disulfide bonds and multiple hydrogen bonds within the polymer matrix to enhance the self-healing ability. Such bonds can break and reform, enabling the material to heal at room temperature. The quadruple hydrogen bonds in the side chains of PUs facilitate rapid, dynamic reconstruction, contributing to excellent self-healing properties [123,132,135].
(c) Interfacial Interactions: The mechanical strength and self-healing efficiency could be improved with strong interfacial interactions, such as hydrogen bonding between graphene oxide nanosheets and the PU matrix. Moreover, the parallel arrangement of graphene within the PU matrix enhances impermeability and corrosion resistance, properties that are of great importance for maintaining material integrity in harsh environments [123,132,135].
(d) Microcapsules and Healing Agents: Some nacre-inspired materials use a microencapsulation of healing agents that breaks during the growth of a crack and releases the healing agent into the crack. The polymerisation of this healing agent thus heals the material. Others include UV-curable resins embedded in the unit cells, which enable repeated healing by capillary action and upon exposure to UV light, thereby making the material self-heal multiple times [136,137,138].
(e) Nanoparticle Migration: The polymer-grafted nanoparticles in the material can migrate to and accumulate at the faces of existing microcracks. This self-healing functionality, through crack filling and repair, further enhances the material’s durability and resilience [134,139]. In self-healing polymer nanocomposites, nanoparticles induce interfacial reconstruction via multiple covalent and non-covalent interactions, leading to an improved mechanical strength and self-healing capability [140]. In nacre-inspired materials, the nanoparticles can migrate to damaged areas, driven by various interactions such as electrostatic forces, to facilitate repair [141]. This process is analogous to that in biological systems, in which cells migrate to injury sites to promote healing [142].

In 2019, Gaolai Du et al. [133] developed a nacre-inspired composite material with self-healing and shape-changing capabilities. This material incorporates a dynamic covalent polymer network that facilitates self-healing in a rigid, high-filler structure through reversible Diels–Alder chemistry. Important features of this composite include the ability to be programmed into different permanent and temporary shapes via plasticity and shape memory effects, respectively. Healing occurs in the polymer matrix and at the polymer–filler interface. Healing via thermal treatment at 120 °C restores chemical bonds and mechanical properties. Notably, the composite can self-heal damage without a specific size limit, recovering up to 96% of its initial strength. The healing efficiency depends on factors such as bending strain, fatigue cycles, and the mobility of ceramic fillers within the polymer matrix. Extensive mechanical testing confirmed that the composite can recover its properties after damage, demonstrating its practical utility. This process, although relatively easy and scalable, shows promise for incorporating different fillers to achieve complex functionality. The diverse healing mechanisms indicate that the developed material can serve as a durable, tough structure with an autonomic self-healing capability against internal damage—a goal of materials science and bioinspired engineering. The general strategies for synthesising nacre-inspired materials include layer-by-layer deposition techniques, additive manufacturing, and the use of organic scaffolds that control the shape and orientation of inorganic components [143,144,145]. These methods have enabled the production of materials with enhanced mechanical properties, such as increased strength, toughness, and strain-to-break [103,146]. Advanced modelling techniques such as the eXtended Finite Element Method (XFEM) and phase-field models have been employed to simulate the mechanical behaviour of nacre-inspired composites. These models help optimise the design of materials for specific applications by tuning microstructural parameters [147,148].

##### Nacre-Inspired Hydrogels

Nacre-inspired hydrogels are mineralised, multi-crosslinked hydrogel membranes with a “rigid silica in soft polymer” nacre-inspired structure, exhibiting superior tensile strength (4.1 ± 0.08 MPa), excellent pure water flux, and a 99% oil/water rejection rate, along with remarkable superhydrophilicity and underwater superoleophobicity. The addition of SiO_2_ nanoparticles enhances the anti-swelling properties, roughness, and hydrophilicity of these membranes, thereby improving their effectiveness in separating water-soluble organic pollutants and dyes, potentially for use in water purification. The resultant hydrogels are biocompatible and mechanically robust, and therefore suitable for biomedical applications in drug delivery and tissue engineering. These nacre-inspired hydrogels possess significantly enhanced mechanical properties and functionality compared to their traditional counterparts [149,150].

##### Nacre-Inspired Energy-Absorbing Materials

Nacre-inspired energy-absorbing materials exhibit exceptional mechanical properties owing to a hierarchical architecture, ranging from brick-like patterns to layer waviness and ultimately to interlocking interfaces. The singular structure enables a combination of localised energy absorption, stiffness, toughness, strength, and light weight, resulting in a remarkable durability and resilience [105]. Mimicking the design principles of nacre enables the engineering of nacre-inspired nanocomposites that, through a photothermal energy cascade, can control their mechanical properties as they approach adaptive steady states that balance energy uptake and dissipation [128].

##### Thermal Management Materials

Nacre-inspired composites exhibit a remarkable multifunctionality owing to the combination of features such as high thermal conductivity, mechanical strength, and electrical insulation, making them suitable for advanced applications. The composite film made of BNNS-chitosan attained a value of thermal conductivity of 26.3 W/(m·K) [151]. Similarly, another material was a BNNS-based nacre-inspired film with an in-plane thermal conductivity of 48.5 W/(m·K) [152]. These materials also retain excellent mechanical properties, including a 104.5 MPa tensile strength and an 8.7 GPa Young’s modulus for the BNNS-chitosan composite film, and a 259 MPa tensile strength for a composite with phenylphosphonic acid and graphene nanoplatelets. In addition, they exhibit an outstanding electrical insulation, as reflected by the BNNS-chitosan composite film [153]. Also, the composite containing carbon nanotubes and graphite nanoplatelets, in addition to a high thermal conductivity, shows a very significant electrothermal and electromagnetic interference shielding performance. These properties make nacre-inspired materials highly valued in electronic devices, even outperforming commercial silicone pads in cooling LED chips [154], while MXene nanocomposite papers exhibit a high thermal conversion efficiency for applications in wearable devices that require EMI shielding and thermal management [155]. Furthermore, they are suited for high-power electronics such as smartphones and LED modules, with the phenylphosphonic acid and GNP composite film giving a better cooling performance compared to commercial tinfoil [153].

##### Nacre-Inspired Sensors and Actuators

The nacre-inspired devices are realised via the self-assembly of materials and properties, including intercalated MXene nanocomposite films, carboxymethyl cellulose, MXene nanosheets, and multivalent aluminium ions. Unlike traditional sensors and actuators, these nacre-inspired devices enable multiple functions with several advantages, such as wearable epidermic sensing over a wider range, faster response and recovery times, and multiple-stimulus response actuation that is not attainable with conventional devices. This implies that they achieve an outstanding sensing performance and actuating ability, ultrahigh mechanical strength, desirable toughness, and impressive wet tensile strength at high humidities. Such biomimetic sensors and actuators will possess very promising applications in robotics and biomedical engineering, including healthcare monitoring, intelligent soft robots, smart switches, human–machine interfaces, artificial muscles, and generators. They can also be fabricated to mimic applications such as flying dragonflies, human finger manipulation, artificial muscles, and moisture-electric generators [156,157,158,159].

##### Optical and Photonic Materials

Naturally translucent and birefringent, nacre is analysable through optical polarimetric techniques [160]. It also exhibits inherent iridescence with a characteristic microstructural arrangement, which is used in the synthesis of materials with similar optical property replication capabilities [161]. Further inspired by nacre, great advances have recently been made in the preparation of 2D photonic nanostructures comprising graphene and TiO_2_ nanosheets. These biomimetic photonic structures exhibited an excellent interlayer charge transfer and markedly enhanced light-response behaviour, which is ideal for solar energy-harvesting and conversion devices [162]. In addition, nacre-inspired materials also enable the construction of nanofluidic devices for photo-actuated directional ion transport, which may be based on structural asymmetry [163]. In addition, hybrid films with a nacre-inspired microstructure, prepared with amino clays and carboxylated cellulose nanofibrils, show an excellent visible light transmittance with superior mechanical properties, which are promising for optoelectronic applications [164].

#### 2.5.7. Nacre-Inspired Energy Applications in Batteries

**Figure 7:**
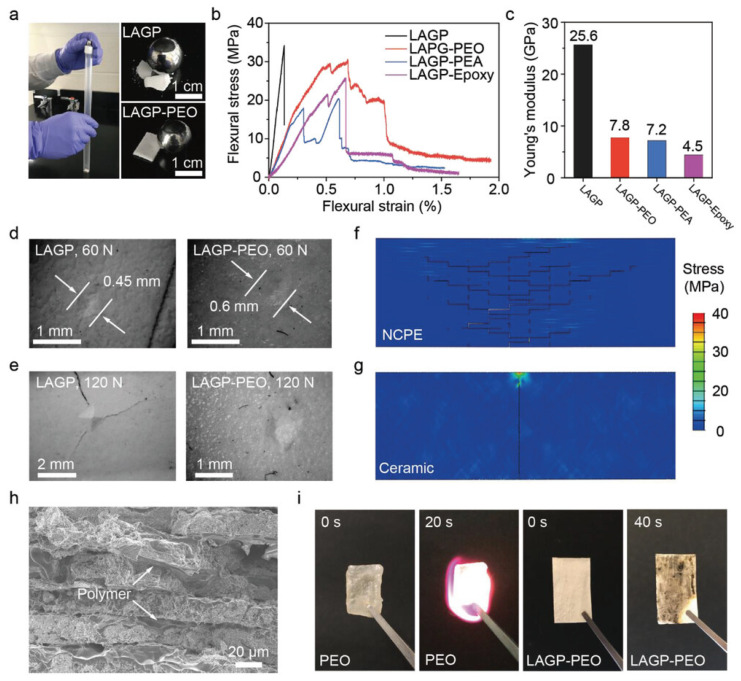
Mechanical properties of NCPEs and toughening mechanisms. (**a**) Impact test of as-fabricated LAGP–PEO NCPE and pure ceramic films, showing higher impact resistance in an LAGP–PEO NCPE film. The ball dropped from 20 cm for pure LAGP ceramic and from 40 cm height for NCPE, respectively. (**b**) Flexural stress–strain curves of NCPEs and pure ceramic films through three-point bending tests. (**c**) Young’s modulus of NCPEs and pure ceramic. (**d**,**e**) Vickers indentation of pure ceramic film and LAGP–PEO NCPE film using loads of 60 N (**d**) and 120 N (**e**). (**f**,**g**) Nonlinear finite element simulations of (**f**) tortuous crack propagation through interfacial polymer failure in an NCPE film and (**g**) a straight crack in a pure ceramic film under the same force load as in (**f**). (**h**) Fractured surface of LAGP–PEO NCPE showing extensive interfacial failure. (**i**) Optical images of ignition tests on pure PEO and LAGP–PEO NCPE films [165].

In 2020, the contributions to the development of solid-state batteries by Aijun Li et al. [165] were immense because, whereas previously there had been conceptions of nacre-inspired solid composite electrolytes based on a “brick-and-mortar” microstructure, there were combinations of ceramic and polymer materials that assured an enhanced mechanical strength and flexibility, usually characterising traditional electrolytes as shown in Figure 7. Accordingly, the nacre-inspired ceramic/polymer electrolyte (NCPE) presented a much higher fracture strain of 1.1% compared to that of pure ceramic electrolytes at 0.13% and a significantly higher ultimate flexural modulus of 7.8 GPa compared with pure polymer electrolytes at 20 MPa, which signifies a substantial improvement in the mechanical properties crucial for durability and reliability. In addition, compared with pure ceramic or polymer electrolytes, the electrochemical performance of NCPE is much better, especially under mechanical load. The pouch cell with LAGP/poly(ether-acrylate) NCPE shows steady cycling with a capacity retention of 95.6% over 100 cycles, even under a large point load of 10 N. Moreover, composite electrolytes are designed to prevent lithium dendrite growth in lithium-metal batteries that can trigger thermal runaway and failure; the hard ceramic and tough polymer layers work in concert to mitigate this risk. These findings open new frontiers in the development of solid-state lithium-metal batteries and structural energy storage solutions, as the proposed NCPE design could make energy storage devices safer and more efficient. Comprehensive experimental data, including mechanical tests and electrochemical performance evaluations, further substantiate the advantages of the proposed design relative to existing materials. During the same period (2020), Seunghyeon Lee et al. [166] produced montmorillonite/DMSO nanocomposites mimicking the nacre structure. This material exhibits outstanding properties, including a very high ionic conductivity (~2 × 10^−4^ S cm^−1^ at room temperature), an excellent mechanical strength of 55.3 ± 4.8 MPa, and a wide thermal stability from −100 to 120 °C. Such characteristics make the material highly suitable for use in solid-state batteries, addressing both the safety and performance challenges associated with liquid electrolytes.

In 2022, Jizhang Chen et al. [167] presented the preparation and properties of a nacre-inspired composite film using Ti_3_C_2_T_x_ and nanofibrillated cellulose (NFC). Among promising novel electrode materials for supercapacitors is Ti_3_C_2_T_x_; however, it exhibits restacking and numerous fluorine-rich functional groups that impede its electrochemical behaviour. In this respect, the authors modified delaminated Ti_3_C_2_T_x_ flakes through alkalisation and post-annealing, thereby removing the impediment posed by -F and -OH functional groups to electroactivity. Modified Ti_3_C_2_T_x_ was incorporated into an NFC matrix derived from soybean stalks; the composite exhibited improved mechanical properties, thereby preventing the dense packing of Ti_3_C_2_T_x_ and enhancing electrolyte ion transport. This strategy produced a composite film that, in addition to having excellent mechanical properties characterised by a 53.9 MPa tensile strength and a high electrical conductivity of 24,930 S·m^−1^, ensures a superior electrochemical performance in supercapacitors and zinc-ion capacitors. In particular, the composite exhibited high capacitances of 303.1 F g^−1^ at 1 mÅ cm^−2^ and 211.4 F g^−1^ at 10 mÅ cm^−2^ with excellent cycling stability in a quasi-solid-state supercapacitor configuration, maintaining 92.84% of its capacitance after 10,000 cycles. Their work represents an effective approach to fabricating high-performance flexible electrodes that can be extended to other energy storage devices based on two-dimensional materials.

## 3. Nacre Definition and Composition

Nacre is an inner shell layer in molluscs that provides protection against predators and mechanical forces, characterised by both high strength and toughness [111]. Its toughness is three orders of magnitude higher than that of the mineral it constitutes, making nacre a biomimetic model material for designing new composite materials. Furthermore, nacre exhibits a species-specific ultrastructure that is believed to result from adaptations to diverse environments. Nacre has been common in mollusc shells since the Ordovician (450 million years ago) and is abundant and well preserved in the fossil record [81,168].

### 3.1. Nacre Appearance

The term “nacre” is conventionally used to refer to the iridescent inner layer in the shells of many molluscs, such as oysters and abalones. Nacre is commonly white to bluish in colour, with an effect that is typically both iridescent and lustrous due to its microscopic structure—the aragonite platelets. This optical effect results from the interference of light waves reflecting off layers within the nacre [81,101,169,170]. Nacre is iridescent and lustrous due to its microstructure and the optical interactions of its layered composition. Such unique properties have made nacre a popular subject of study in natural observation and biomimetic material design. The iridescence in nacre is mainly based on its one-dimensional photonic crystal structure. Its scientific basis involves reflecting light at different wavelengths, which causes it to appear in typical rainbow-like colours [171,172]. The thickness of the aragonite layers is a key factor in this optical phenomenon since changes in the latter cause photonic bandgap shifts and, hence, other colours [172]. While most of the light is transmitted through the nacre aragonite plates, the reflected portion is wavelength-selective depending on the plates’ thickness. This is the origin of iridescent colours from nacre [171]. In addition, the shining properties of nacre depend on reflectivity and the crystalline microstructure. The reflectivity is so dependent upon the smoothness and compactness of the layer of nacre due to porosity, as higher porosities cause surface imperfections, which diminish lustre. The thickness of the nacreous layer and the consistency of the crystal thickness of each aragonite crystal tablet are also crucial; a thicker layer and consistent crystal thickness improve the resulting interference colour and, therefore, improve appearance, while the variation in crystal thickness weakens the interference colour [173,174,175].

### 3.2. Nacre Formation

**Figure 8:**
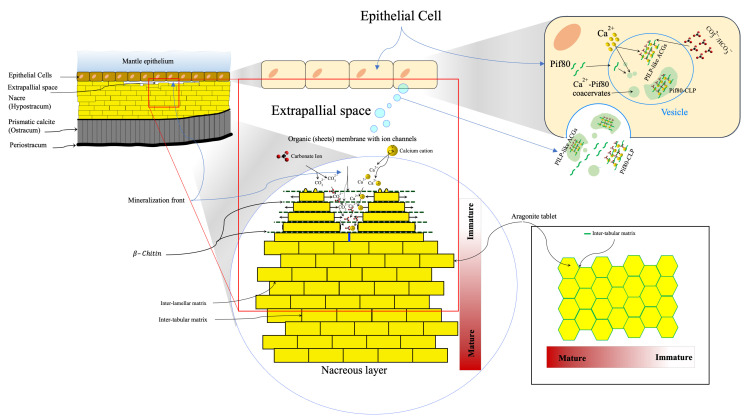
Hierarchical organisation of the mollusc shell nacreous layer: mineralisation front and the “brick-and-mortar” assembly of aragonite tablets within the organic framework. The mantle epithelium secretes (Ca^2+^) and carbonate ions via vesicles into the extrapallial space. These precursors, along with organic components such as Pif80 and β-chitin, form a mineralisation front. The diagram details the transition from immature to mature aragonite tablets, organised by inter-lamellar and inter-tabular matrices into a highly ordered “brick-and-mortar” microstructure. This has been modified from its original form in *Science* and *RSC Advances* journals, and the authorisation for this change was obtained [82] and [182].

Nacre formation is a highly regulated process involving interactions between organic matrices and mineral components. Specifically, the organic matrix within the proteins contributes to the directed nucleation and growth of aragonite crystals. The formation of nacre may be influenced by environmental conditions such as temperature, which can modify the thickness of the nacre layer. It includes biomineralisation and key aspects of materials science. Nacre has a complex hierarchical microarchitecture spanning several orders of magnitude in the length scale, including columnar architectures, mineral bridges, and nanoasperities, which are responsible for its remarkable combination of stiffness and strength, along with low weight [82]. In the mollusc shell, proteomes control the nucleation and assembly of the nacre tablets from precursor mineral nanoparticles, showing specific proteins involved in nacre formation [176,177]. The organic matrix in nacre consists of β-chitin and silk fibroin-like proteins, which play an important role in biomineralisation (Figure 8). These components control the size and morphology of the aragonite crystals [178,179]. These membranes form early in the process of nacre formation and help organise the deposition of calcium carbonate layers, acting as a scaffold for subsequent mineralisation [180,181].

#### 3.2.1. Aragonite

Amorphous calcium carbonate (ACC) acts as a precursor in the biomineralisation process, transforming into aragonite, which is the crystalline form of calcium carbonate found in nacre [183,184,185,186]. ACC precursors have been observed at the growth front of nacre in gastropod shells [184]. The aragonite in nacre is a crystallographic form of CaCO_3_ [82]. The transformation of ACC to aragonite involves a complex process regulated by environmental conditions, organic molecules, and ions such as magnesium (Mg^2+^) [187,188,189,190]. Proteins and polysaccharides in the organic matrix of nacre impact the ACC, stabilising and guiding its transformation into aragonite [186,191,192].

##### Aragonite Nucleation and Growth

Nacre biomineralisation is characterised by complex mechanisms of nucleation, growth, and polymorph selection. Aragonite crystals, the main mineral constituent of nacre, are nucleated and grown in an organic matrix composed of proteins and chitin. These organic components play an important role in controlling the nucleation, growth, and polymorph specificity of the aragonite crystals [193,194]. In addition, proteins and chitin provide structural frameworks that control the transformation of amorphous calcium carbonate to aragonite [183,195]. The main characteristic of these biomineralisation processes is that a particular polymorph is selected; this arises from specific proteins in the organic matrix, which can promote the formation of aragonite rather than calcite (Table 1).

##### Mechanisms of Transformation

The phase transformation of ACC to aragonite in nacre is complicated; it represents the influence of different factors and mechanisms. Environmental factors, such as temperature and the presence of special ions like Mg^2+^, together with organic molecules, are of great importance in determining the stability of ACC and its crystallisation pathway [187,188,189,196]. Nacre’s organic matrix is composed of proteins and chitin, which form a significant scaffold for the nucleation and growth of aragonite crystals from ACC [183,184,191]. Several proteins have been identified as playing significant roles in the binding process of ACC during its transformation to aragonite, including paramyosin [186]. It represents a multistep assembly transformation, initiated by the formation of an ACC layer, followed by the growth of aragonite nanostacks and the eventual assembly into hexagonal tablets [197].

##### Aragonite Function in Nacre

The nacreous layer is the innermost layer of many shells. It comprises tiny polygonal aragonite tablets, each 5–15 micrometres wide, arranged in parallel layers 0.5 micrometres thick. Sheets of interlamellar organic matrix separate these layers [57,198,199]. Aragonite formation in nacre occurs through the nucleation and growth of aragonite crystals in a protein matrix, where proteins act as a structural framework, thereby highly influencing the mineralising organic matrix due to the osteoinductive properties of biogenic aragonite. This defines the role of organic materials during the formation process. This indicates an understanding of how nacreous aragonite evolves, as both its nucleation and growth within the inner-shell film system are influenced by matrix proteins, thereby characterising sequential morphologies with information [200].

**Table 1:** Functional attributes and descriptive mechanisms of nacre.

Function	Description	References
Structural Role	Brick-and-mortar structure with aragonite platelets and organic matrix	[201]
Nanostructure	Aragonite platelets composed of nanograins, enhancing mechanical properties	[202]
Biomineralisation Process	Nucleation and growth within an organic matrix, transformation from ACC to aragonite	[203]
Polymorph Selection	Proteins induce aragonite formation over calcite	[204]
Enhanced Toughness	High fracture toughness and energy absorption due to hierarchical structure	[82]
Domain Structure	Specific orientations of aragonite crystals contributing to mechanical strength	[205]

#### 3.2.2. Proteins in Nacre

**Figure 9:**
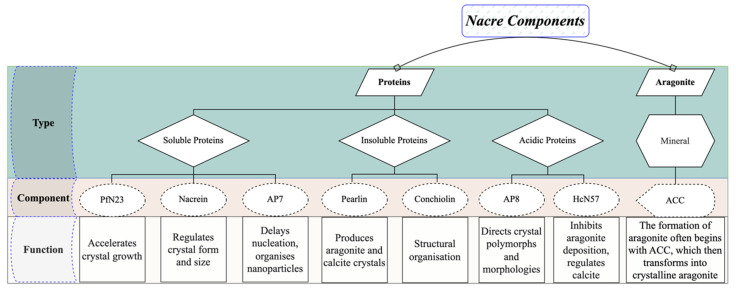
Nacre component and its functions.

Although a number of the organic constituents occur in nacre, proteins remain the major constituents and are associated with the mediation of its very special lamellate structure [193,194,206]. The formation of nacre in mollusc shells is under the control of a complex of proteins [206]. Nacre formation in gastropods involves a vesicular transport system that moves organic and mineral precursors from the mantle epithelium to the mineralisation chamber. The transport occurs in lipid bilayer vesicles through exocytosis from the outer mantle epithelium. The surface membrane is a dynamic structure that grows by adding vesicles to the outer surface and shrinks by forming interlamellar membranes on the inner surface. This membrane is where chitin and protein polymerisation occur, thereby forming the final structure of the interlamellar membranes. These organic matrices transport large amounts of organic calcium into the mineralisation compartment, thereby maintaining a transport system driven by acidic calcium-binding proteins [207]. Molluscan nacre is a fascinating object, and proteins that determine the shape and orientation of individual nacreous platelets in extant gastropods have not changed since the Late Triassic. These unchanged molecular mechanisms underlie the origin and development of gastropod nacre [208]. Several proteins have been identified in forming and regulating aragonite crystals, playing important roles in the biomineralisation process. These proteins can be categorised based on their solubility and functions (Figure 9).

##### Soluble Proteins

Soluble proteins are essential in the biomineralisation process. Several key proteins have been identified in forming and regulating aragonite crystals; among them, the basic protein PfN23 accelerates crystal growth and is essential for the formation of aragonite crystals [209]. Nacrein, in turn, controls form and size during aragonite crystallisation, exerting an inhibitory effect in its free state but adopting a regulatory role regarding crystal formation once bound to the insoluble shell matrix (ISM) [210]. In addition to the aforementioned, AP7 represents an intracrystalline protein capable of inhibiting nucleation before arranging mineral nanoparticles into ordered clusters [211]; furthermore, testifying to the multi-faceted and complex involvement in the processes of biomineralisation, there is the input of soluble proteins.

##### Insoluble Proteins

Pmaz-pearlin, also referred to as Pearlin, is an insoluble protein found to be important for biomineralisation and is presumed to form aragonite and calcite crystals in vitro. Within Pearlin, multiple acidic regions with conserved cysteine residues confer stability [212]. Another major insoluble protein found within nacre is conchiolin. Conchiolin is said to appear in a network fashion in the nacre matrix, constituting the network that underlies the structural organisation of nacre and providing a scaffold upon which mineralised components are assembled [213]. The sum of these insoluble proteins illustrates, in a complex way, the interplay between organic and inorganic components in the development of biomineralised tissues such as nacre, and highlights the sophisticated biological processes by which materials with exceptional mechanical properties are formed.

##### Acidic Proteins

Acidic proteins are important in the biomineralisation process of calcium carbonate structures. An example is AP8, a highly acidic protein that binds calcium ions and significantly influences the morphology of CaCO_3_ crystals, thereby guiding crystal polymorphs and morphologies [214]. Silk-like proteins, including HcN57, which contain aspartic acid residues, have also been found to play multiple roles in biomineralisation. These proteins inhibit aragonite deposition, control calcite morphology, and are responsible for nacre nucleation [215]. The action of these acidic proteins in concert reinforces the conclusion that organisms exert elaborate and specific control over the mineralisation process, further supporting the conclusion that biological crystal growth processes are complex and highly developed.

#### 3.2.3. Proteins Function in Nacre

The hierarchical structure of nacre is developed through the complex regulation of many different proteins, each with specific roles in the nucleation and growth of aragonite crystals [209,210]. Proteins such as PfN23 and nacrein are involved in controlling the nucleation and growth processes, which are important for the proper development of the unique structure of nacre [209,212,213]. Conchiolin and Pearlina are some examples of proteins involved in maintaining structural integrity and in the organisation of the nacre matrix, while AP7 and AP8 act directly with the mineral nanoparticles currently forming to regulate early nucleation steps that determine the morphology of the final crystals [211,214,216]. The specific functions of all these protein–mineral interactions must be expressed in concert if the characteristic properties and structure of nacre are to be realised.

## 4. Hierarchical Architecture and Multiscale Organisation of Abalone Nacre

**Figure 10:**
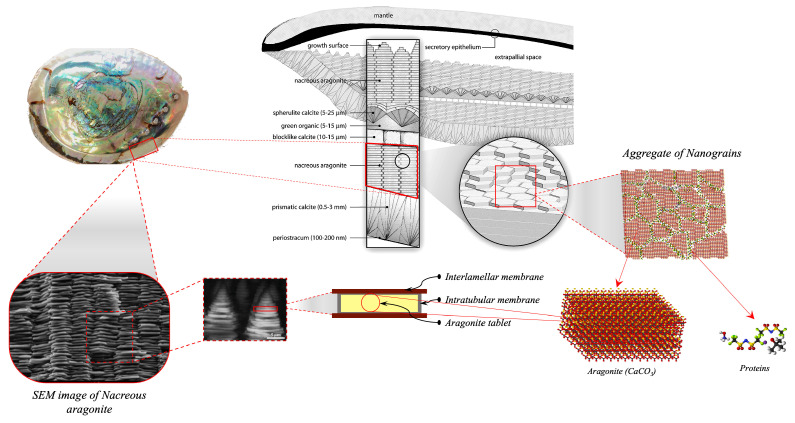
Schematic of the red abalone (gastropod) molluscan shell anatomy, showing a vertical section of the outer edge of the shell and mantle with an enlargement indicating the thickness dimensions of the shell structures. In addition, the hierarchical structure of nacre ranges from nano- to micro-, to meso-, to structural length scales. This has been modified from its original form in *RSC Advances* and John Wiley and Sons journals, and the authorisation for this change was obtained from [82] and [218].

The structural complexity of the abalone shell, as shown in Figure 10, results from a biologically controlled mineralisation occurring in the extrapallial space between the mantle and the growth surface of the shell [6]. The secretory epithelium facilitates the deposition of various mineral layers, beginning with the outermost periostracum (100–200 nm) and the robust prismatic calcite (0.5–3 mm). The transition to the inner shell involves distinct morphological zones, including block-like calcite (10–15 μm), a green organic layer (5–15 μm), and spherulite calcite (5–25 μm), which eventually give way to nacreous aragonite [217]. The SEM image of nacreous aragonite reveals a highly ordered “brick-and-mortar” microstructure where individual aragonite tablets are stacked into lamellae. This mineral phase is reinforced by an organic matrix composed of the interlamellar and intratubular membranes, which arrest crack propagation and provide elasticity. At the nanoscale, these tablets are shown to be an aggregate of nanograins, fundamentally composed of the crystalline lattice of aragonite (CaCO_3_) and specialised proteins that direct the assembly and orientation of the biocomposite [82].

## 5. Nacre Structure and Mechanical Properties

Nacre is a “brick-and-mortar” structure with oriented, plate-like aragonite crystals and organic constituents, exhibiting remarkable mechanical properties due to this structure [219,220]. The organic matrix, which constitutes only 5% of the volume in the nacre, plays an integral role in its formation and mechanical behaviour, responsible for its exceptionally high fracture toughness and energy dissipation properties [219,221]. Sliding, pullout, crack deflection, and interface separation are involved in the microscale mechanical behaviour of nacre, accounting for the high fracture toughness of the material [221]. The hierarchical organisation and specific structural features in nacre, such as the interlocking structure of aragonite tablets and organic layers, determine the extraordinary mechanical properties and stimulate biomimetic strategies in materials science and nanotechnology [219,222]. This hierarchical structure endows nacre with extraordinary mechanical properties, such as a high strength and toughness, which are significantly greater than those of its individual components [101,130,144,219,223,224].

Nacre has a complex hierarchical microstructure across multiple length scales, from nanoscale to macroscale, comprising columnar structures, sheet tiles, mineral bridges, and polygonal nanograins [82,111,221]. Thus, in nacre, aragonite platelets are arranged in layers and tightly stacked to form a three-dimensional brick-wall structure, with the mortar being a thin layer of biopolymers [225,226]. This ingenious structure provides nacre with its very high toughness, significantly higher than that of monolithic aragonite, due to mechanisms such as crack deflection, plastic micro-buckling, and the interlocking of aragonite platelets [82,111,221]. Moreover, the organic matrix further enhances the energy absorption capability of nacre [84,90], making it damage-tolerant while still offering exceptional strength and stiffness for a material composed primarily of brittle ceramic [111,225,227]. Research on nacre has improved our understanding of the biomineralisation process, showing how some natural materials achieve remarkable properties through complex structural organisation. This knowledge also guided the development and synthesis of biomimetic materials that reproduce these natural processes [228,229]. Inspired by nacre, researchers have developed various synthetic materials that mimic its structure and properties. These nacre-inspired materials have potential applications in fields such as aeronautics, civil engineering, and biomedical engineering [101,103,116,130,144,219,223,224]. The term “nacre-inspired” refers to materials and structures that mimic the unique properties and hierarchical architecture of nacre, also known as mother of pearl. Nacre is a natural composite material found in mollusc shells, composed primarily of aragonite (a form of calcium carbonate) and organic polymers arranged in a “brick-and-mortar” structure [144,220,230].

### 5.1. Nacre Structure: A Multiscale Perspective

**Figure 11:**
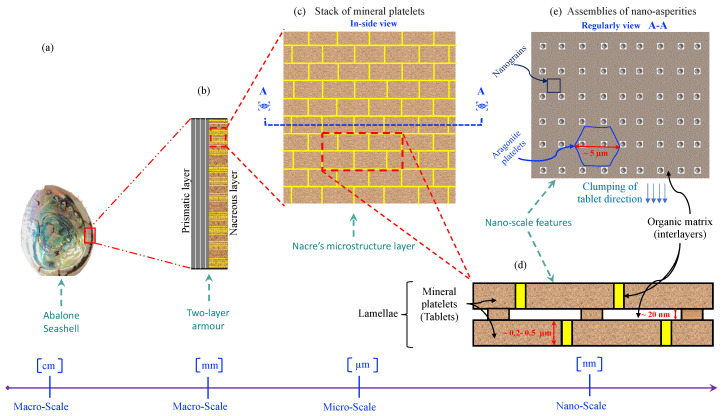
Nacre hierarchical structure from macroscale to nanoscale: (**a**) Abalone seashell; (**b**) The protective armour system; (**c**) The bricks and mortar microstructure; (**d**) Staggered overlapping multi-layer arrangement with mineral bridges (out-of-plane) and adhesive (in-plane) connections between adjacent tablets; (**e**) A polygonal layer with cohesive bonds between adjacent tablets and nano-sized asperities indicated, adapted from [234].

The hierarchical structure of nacre refers to the unique arrangement of building blocks that contributes to its exceptional mechanical properties [82,231]. Nacre has a complex hierarchical microarchitecture that spans multiple length scales and is organised across multiple hierarchical levels, from macro- to nanometre scales. The hierarchical organisation of nacre, from nano- to mesoscale, is regulated by the interaction between proteins and minerals. This structured organisation is pivotal for nacre’s mechanical strength and optical properties. The transition from amorphous calcium carbonate (ACC) to the stable aragonite polymorph involves complex biochemical processes, wherein proteins play an important role in stabilising ACC and enabling its subsequent transformation [232,233]. This structure arises from the assembly of different-sized building blocks during natural growth, as shown in Figure 11. The horizontal bar at the bottom spans from centimetres (cm) to nanometres (nm), visually indicating the scale transition from the macroscale view of the whole shell to the nanoscale features of the nacre structure:

#### 5.1.1. Macroscale

Figure 11a shows an abalone shell at the macroscale, revealing its outer surface and inner iridescence, which are characteristic of nacre. This constitutes the initial basis for the detailed investigation that follows:

##### Intermediate Macroscale

The magnified view of the abalone seashell at the macroscale (mm) in Figure 11b demonstrates the two-layer armour in nacre. It shows two distinct layers, the prismatic layer and the nacreous layer, which constitute this natural material’s overall structure [234,235]. This two-layer armour system protects the abalone shell’s inner soft tissues (the animal) from the environment and predators. The shell’s brittle outer layer (external calcite-based layer) absorbs the initial impact but is prone to cracking. To prevent these cracks from catastrophically propagating through the shell to the animal itself, the nacreous layer (internal aragonite-based layer) is surprisingly robust and tough, with outstanding crack-arresting properties. This nacreous layer, composed of alternating layers of calcium carbonate monocrystal and soft organic material, acts as a lining to maintain the shell’s integrity in the event of the cracking of the outer layer. The unique microstructure and composition of this nacreous layer contribute to its exceptional mechanical properties, making the overall shell structure highly resistant to fracture and damage [236,237]. The crack propagation in nacre’s aragonite platelets differs from pure geologic aragonite, with nacre exhibiting an intergranular mechanism and geologic aragonite a cleavage mode. The nanoparticle architecture and biopolymer interlayer in nacre enhance toughness by deflecting cracks and resisting sliding. This structure allows nacre to resist crack propagation and promote deflection along the biopolymer interface [238]. Understanding the impact of initial flaws in nacre is crucial for comprehending its failure mechanisms and developing nacre-inspired materials with improved mechanical properties [239].

#### 5.1.2. Microscale

An examination of the microscale region of the nacreous layers, with a zoom-in, reveals the intricate microstructure of nacre, characterised by a stacked arrangement of mineral platelets (Side View). The mineral platelets in the nacre microstructure layer are arranged in a stacked, brick-like pattern reminiscent of a meticulously constructed brick wall held together by mortar (Figure 11c) [240,241].

#### 5.1.3. Microscale and Nanoscale

Figure 11d,e shows a detailed, enlarged, partial view of nacre that spans both the micro- and nanoscales of assemblies of nanoasperities (in Regular View). It shows how aragonite platelets (a form of calcium carbonate, CaCO_3_, about 95 wt.%) are organised at the microscale, with nanograins dispersed throughout the structure at the nanoscale. The organic matrix layers (about 5 wt.%) interspersed between the mineral platelets are crucial to the toughness and resilience of the nacre microstructure [102,242,243,244,245,246].

#### 5.1.4. Nanoscale

The inner layer of mollusc shells consists of aragonite lamellae sandwiched between organic layers [247,248,249]. Figure 11e shows these lamellae, individual mineral platelets (tablets) and their spacing at the nanoscale. The spacing between platelets is approximately 0.2 to 0.5 micrometres (µm), with organic matrix layers (~20 nanometres thick) interspersed between them. Each lamella comprises one organic layer between two mineral platelets. The mineral platelets are thin, flat layers composed primarily of aragonite, a crystalline form of calcium carbonate (CaCO_3_), typically 0.5 µm thick. The organic matrix is a thin, thick, interspersed layer of organic materials, such as proteins and polysaccharides, of about 20 nanometres (nm). In another type of nacreous material, the single nacreous crystals of *Pinctada martensii* and *Elliptio complanatus* are composed of small blocks approximately 0.2–0.5 µm wide and 1 µm long. These blocks are generally elongated along the a-axes with curved edges. Occasionally, a small block, or part of it, is divided into units 200–500 Å wide [198]. This matrix acts as a flexible binder, holding the mineral platelets together. This repeated structure creates a “brick-and-mortar” pattern, in which the mineral platelets act as bricks and the organic matrix as mortar. This layering enhances the structural integrity and mechanical properties of nacre. The growth behaviour of abalone nacre is characterised by the clumping of microscopic platelets, which align due to interfacial forces during the intricate biomineralisation process [182]. The organic matrix is important for controlling the precise placement and orientation of these platelets, ensuring tight and well-organised stacking [250,251]. The interface between the mineral platelet and the organic layer of nacre is an irregular micro-topological structure that increases the sliding resistance of the platelets, enhancing fracture toughness [252]. The hierarchical structure, with the clumping of mineral platelets at the nanoscale and the organic matrix, significantly contributes to the toughness and resilience of nacre. The thickness of nacre tablets, which contributes to their iridescence and fracture resistance, is influenced by environmental factors, including temperature. This would imply a biophysical mechanism that integrates environmental cues into the biomineralisation process [175]. This structure enables nacre to withstand impacts and resist fracture, making it an effective natural armour for the mollusc. The nacre structure extends across multiple scales, from the whole shell to the nanoscale arrangement of mineral platelets and organic layers. The hierarchical structure of nacre enables it to exhibit a remarkable combination of strength and toughness, achieved through toughening mechanisms such as plastic deformation, crack deflection, and mineral bridges at the micro- and nanoscale [253].

### 5.2. Nacre Mechanical Properties

Nacre’s distinctive brick-and-mortar microstructure is composed of aragonite platelets bonded by an organic matrix, resulting in an exceptional combination of stiffness, strength, toughness, and fracture resistance that far surpasses its individual constituents [231,254,255,256], as shown in Table 2. Its mechanical properties include a Young’s modulus of 60–90 GPa parallel to the platelets, a shear modulus of about 10 GPa, a tensile strength ranging from 35 to 220 MPa, a fracture toughness between 3.7 and 7 MPa·m^½^ (up to ~30 times that of monolithic CaCO_3_), and a work of fracture of 1.24–1.65 kJ/m^2^, which is roughly 3000 times higher than monolithic CaCO_3_ [101,257,258]. Synthetic nacre analogues can achieve bending strengths of around 91 MPa, and structural features such as tablet waviness can increase the elastic modulus by 23%, strength by 65%, and toughness by 42% [227,259]. Nacre’s remarkable performance arises from synergistic toughening mechanisms (such as crack deflection, platelet pull-out, interfacial sliding, and viscoplastic deformation of the organic phase) that dissipate energy and prevent catastrophic failure [111,256]. Hydration significantly influences its behaviour, with wet nacre exhibiting ductile, plastic deformation, whereas dry nacre behaves in a brittle manner [101,258]. Its hierarchical architecture, incorporating mineral bridges, wavy interfaces, and nanoscale asperities, enables a rare strength–toughness synergy, where increasing structural hierarchy slightly reduces strength but greatly enhances toughness [111,231,254]. Mechanical properties also vary with the loading direction, rate, and environmental conditions [4,101]. This unique balance of stiffness comparable to engineering ceramics, toughness orders of magnitude higher than its mineral constituent, and high strength despite a soft organic phase makes nacre an important model for the design of bioinspired composites with potential applications in aerospace, automotive, and protective materials [111,255,260].

#### 5.2.1. Applications of Nacre’s Mechanical Performance

Nacre’s exceptional mechanical performance (high stiffness, strength, toughness, and impact resistance) has inspired a wide range of applications across engineering, biomedical, protective, and smart material domains. In protective systems, nacre-inspired laminated armour materials are used for bulletproof protection due to their ability to absorb high fracture energy and resist ballistic impacts [261]. In structural engineering, nacre-inspired designs have been applied to engineered cementitious composite (ECC) beams and large-scale cementitious members, enhancing impact resistance, energy absorption, and ductility in infrastructure [262,263]. In biomedical applications, nacre-inspired porous scaffolds and layered composites have been developed for bone repair and dental grafting, offering mechanical properties comparable to those of bone, bioactivity, and tuneable physical characteristics [264]. In lightweight engineering materials, the scalable production of wooden artificial nacres (WANs) has yielded high bending, tensile strength, and toughness at a low density, making them promising for aerospace and automotive structures [103]. In smart materials and electronics, nacre-inspired nanocomposites have been integrated into shear-stiffening gels for personal protection and multifunctional electronics, as well as responsive devices such as strain sensors, displays, artificial muscles, and robotics [129,265]. Additionally, nacre-inspired composites with superior damping performance are being explored for vibration control in high-performance mechanical systems [266]. These applications leverage nacre’s hierarchical brick-and-mortar architecture, which enables a rare synergy of strength and toughness, making it a versatile model for designing advanced materials across diverse fields.

#### 5.2.2. Industries Benefiting from Nacre’s Mechanical Performance

Nacre’s exceptional mechanical properties enable its use across multiple industries, often through bioinspired composites that mimic the hierarchical “brick-and-mortar” nacre structure, such as:

- **Protective Equipment and Defence:** Nacre-inspired laminated armour materials are applied in bulletproof protection, offering high fracture energy absorption, impact resistance, and toughness for military and law enforcement gear [261,267]. Camouflage-capable nacre-mimetic ceramics also serve to protect radar and communication equipment [267].
- **Aerospace and Automotive:** Inverse nacre-inspired epoxy–graphene nanocomposites and 3D-printed nacre structures improve fracture toughness, crack resistance, and impact performance in lightweight structural components for aircraft, automobiles, and wind devices [93,268,269,270].
- **Biomedical:** Nacre-inspired hydroxyapatite/polymer composites and silk fibroin nacre are developed for load-bearing bone implants, dental grafts, and other biocompatible devices, combining mechanical robustness with bioactivity and osseointegration potential [271,272].
- **Sports and Anti-Collision Systems:** Layered bulk composites with nacre-inspired structures show a high impact resistance and protective warning functions, suitable for sports equipment and safety gear [269].
- **Structural and Civil Engineering:** Nacre-mimetic designs enhance strength and toughness in engineered cementitious composites and other infrastructure materials, improving ductility and energy absorption under load [224,273].
- **Electronics and Smart Materials:** Conductive nacre-inspired nanocomposites enable the self-monitoring of structural integrity, with potential in multifunctional electronics, sensors, and robotics [268].
- **Advanced Materials Manufacturing:** The scalable production of nacre-inspired graphene oxide-based bulks and mineralised materials supports high-performance applications requiring a tailored strength–toughness balance [270,274].

## 6. Evolution from “Nacre” to “Nacre-Inspired” Materials

Nacre is renowned for its exceptional mechanical properties, such as high strength and toughness, which are attributed to its hierarchical “brick-and-mortar” structure [101,130,143,219,223]. It is composed of microscopic aragonite platelets arranged in a brick-and-mortar pattern, bound together by organic proteins. This hierarchical structure enables nacre to absorb and dissipate energy, rendering it highly resistant to fractures [141,142,143]. The study of nacre formation can also contribute to the development of sustainable materials, as it relies on natural, biodegradable, and renewable resources [275]. By mimicking the biomineralisation process, researchers can create materials that are more environmentally friendly and have a reduced environmental impact. Moreover, the complex interplay between the inorganic aragonite platelets and the organic matrix results in a far stronger and tougher material than its individual constituents. Inspired by nacre’s extraordinary properties, researchers have developed synthetic materials that mimic its structure and performance. The transition from studying natural nacre to creating nacre-inspired materials: Scientists began analysing the microstructure of nacre using advanced imaging techniques, including scanning electron microscopy (SEM) [276,277] and transmission electron microscopy (TEM) [256,278,279]. Other researchers employed different methods, such as the optical technique known as “hyperspectral interference tomography”, which rapidly and non-destructively extracts nanoscale structural information from nacre in mollusc shells. This method was applied to study nacre from two different species: red abalone and rainbow abalone [280]. By applying boundary conditions to adjacent boundaries of a thin dielectric film and using Malus’ law, Nicole Fan et al. [281] calculated the characteristic matrix, reflection and transmission coefficients, and spectral reflectance for light waves, considering both coherent and incoherent light sources with a model featuring 200 submicroscopic layers. Interference patterns were used to estimate the aragonite tablet thickness in nacreous pearls, showing that thicker conchiolin layers enhance colour chroma while a higher conchiolin index decreases saturation. These patterns remained stable against minor variations and were consistent across different lighting conditions. The observations were conducted with a glass diffuser and a gem microscope; spectra were collected using an MK350N spectrometer; and detailed images were captured with a 90 mm macro lens, using ISO 200, f/32, and Incandescent white balance settings.

These studies revealed the intricate arrangement and composition of the aragonite platelets and organic matrix, yielding a blueprint for synthetic replication. Various fabrication techniques were employed to create nacre-inspired materials. Among these techniques, one approach is layer-by-layer assembly [161,282], in which thin layers of inorganic and organic materials are sequentially deposited to replicate the brick-and-mortar structure of nacre [144,283,284]. Another method uses “3D printing” to construct nacre-inspired composites with tailored properties [93,97,98,99,285,286]. Additionally, biomineralisation processes are explored to grow synthetic nacre-inspired materials by controlling mineral crystallisation in the presence of organic molecules [202,287,288,289].

Artificial materials replicating nacre’s (nacre-inspired) hierarchical structure show impressive mechanical properties, such as exceptional strength and toughness. The fabrication of these materials includes nacre-inspired lamella-structured Ti-Ta composites [290], silk nanofibrils (SNFs) [291,292], hydroxyapatite (HAP) [293,294], chitin nanofibril (CNF) nanocomposites [295], and graphene/poly(vinyl alcohol) composite film [253,296]. Recent advances in producing nacre-inspired nanocomposites via layer-by-layer deposition techniques demonstrate the ability to fabricate finely structured layered materials [297,298]. Nacre-inspired materials demonstrate a remarkable increase in toughness compared to their constituent minerals, with a work of fracture approximately 3000 times higher than that of pure constituent minerals. These materials offer an ideal combination of high strength and fracture toughness, enabling the production of alternative, sustainable high-performance structural and functional materials [101]. These engineered composites exhibit strong, durable properties suitable for diverse applications, owing to the precise control of nanoscale thickness across various disciplines. Scientists and researchers can create advanced materials that combine strength and flexibility by emulating and drawing inspiration from the nacre’s natural design (a hierarchical, brick-and-mortar structure) and its reaction mechanisms (self-assembly, biomineralisation, and layer-by-layer deposition).

## 7. Fabrication Techniques of “Nacre-Inspired” Materials

The current state of the art in fabricating nacre-inspired materials encompasses a broad range of fabrication techniques, each tailored to different production scales and mechanical performance requirements. Rapid in situ mineralisation offers scalable bulk composite formation with hierarchical lamellar structures; 3D printing (including fused deposition modelling and direct ink writing) provides precise microstructural control; and freeze casting, particularly bidirectional freeze casting, represents an environmentally friendly alternative for producing nacre-mimetic composites. Complementary methods such as layer-by-layer assembly and sol–gel synthesis, often combined with interfacial engineering through processes like ALD, have been used to enhance mechanical properties through controlled platelet assembly and strengthened organic–inorganic interfaces. Recent innovative approaches also incorporate multifunctional nanomaterials (such as graphene oxide and MXene) via scalable methods such as magnetic assembly and vacuum filtration, thereby further broadening the potential applications and functional integration of nacre-inspired materials. Table 3 outlines the fabrication techniques for nacre-inspired materials, alongside their mechanical properties and respective advantages and limitations.

### 7.1. Freeze Casting (Ice Templating)

**Figure 12:**
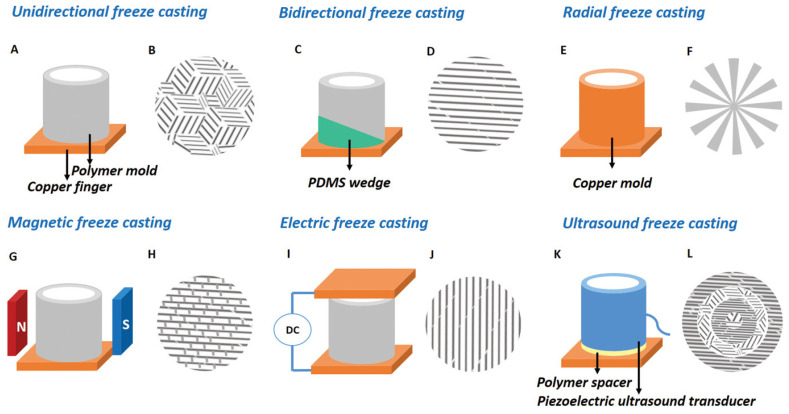
Schematics of freeze casting techniques and corresponding top-view microstructures of resulting scaffolds. (**A**,**B**) Unidirectional, (**C**,**D**) bidirectional, (**E**,**F**) radial, (**G**,**H**) magnetic, (**I**,**J**) electric and (**K**,**L**) ultrasound freeze casting [300].

Freeze casting is a versatile technique for creating laminar porous materials by exploiting the phase transition of solvents to structure materials such as ceramics, metals, polymers, and biomacromolecules (Figure 12) [299,300,301]. This method has been extensively used to fabricate nacre-inspired composites that mimic the “brick-and-mortar” structure of natural nacre, yielding materials with exceptional mechanical properties [302,303]. The process involves several steps, suspension preparation, solidification, sublimation, and post-treatment, with parameters such as the solid concentration and freezing front velocity being essential to determine the final structure and properties [304,305]. Freeze casting has been applied in various fields, including aerospace, healthcare, and environmental applications, due to its ability to produce materials with tailored microstructures and multifunctional properties [306,307,308]. In this method, a water-based ceramic slurry is frozen under a controlled, directional temperature gradient. As the ice crystals grow, they push ceramic particles into concentrated lamellae between the ice plates. Once freezing is complete, the ice is removed via sublimation (freeze-drying), leaving behind a porous, lamellar ceramic scaffold. This scaffold is subsequently infiltrated with a polymer, metal, or glass phase to create a high-strength “brick-and-mortar” composite structure [309]. Recent advancements include the use of magnetic fields to control architectural alignment and the development of sintering-free methods to broaden material selection and applications [302,306]. The technique’s ability to create materials with high strength, toughness, and other functional properties, such as thermal conductivity and electromagnetic shielding, makes it a promising approach for future material design and application [299,300,310]. Synthesising recent findings, freeze casting has emerged as a versatile and cost-effective approach for fabricating nacre-inspired composites, offering a precise microstructural control over lamellar architectures, pore size, wall thickness, and ceramic content through adjustments in freezing parameters, additives, and external fields such as magnetic or electric assistance [311,312,313]. This tunability enables the creation of highly aligned porous architectures and mineral bridges that enhance both strength and toughness, with optimised systems achieving flexural strengths up to 931 MPa and crack-growth toughness exceeding 50 MPa·m^1/2^ [313,314,315]. The method’s adaptability across ceramics, metals, and polymers has led to diverse composite systems including (Al_2_O_3_/Al, B_4_C/Al, SiC/Al, TiC/Al, and zirconia/PMMA), extending its utility to functional applications such as electromagnetic interference shielding [314,316,317,318]. Furthermore, freeze casting is environmentally friendly, requires relatively simple equipment, and is scalable to large structures, thereby avoiding toxic emissions and facilitating straightforward sintering [312,319,320,321]. However, conventional freeze casting often produces directionally aligned lamellae, resulting in anisotropic mechanical properties that are stronger along the freezing direction but weaker transversely [311,321]. Replicating the fine structural features of natural nacre—such as platelet waviness, mineral bridges, and nanomorphology—remains challenging, with most outputs limited to lamellar rather than true brick-and-mortar architectures and ceramic contents far below the ~95 vol% found in nature [240,312]. Processing challenges also arise from poor ceramic–metal wettability, necessitating pressure-assisted infiltration, which increases cost, whereas magnetic field assistance can cause particle agglomeration unless mitigated by surface coatings [240,311]. Performance trade-offs are evident, as high soft-phase content can reduce strength, and inverse nacre structures, while improving toughness, may limit strength gains [322]. Moreover, most studies focus on static loading, leaving dynamic impact-resistance mechanisms underexplored [315]. Freeze casting presents a powerful platform for nacre-inspired material design, but overcoming anisotropy, architectural limitations, and processing constraints is critical for fully replicating the exceptional mechanical performance of natural nacre.

### 7.2. Layer-by-Layer (LbL) Assembly in Nacre-Inspired

**Figure 13:**
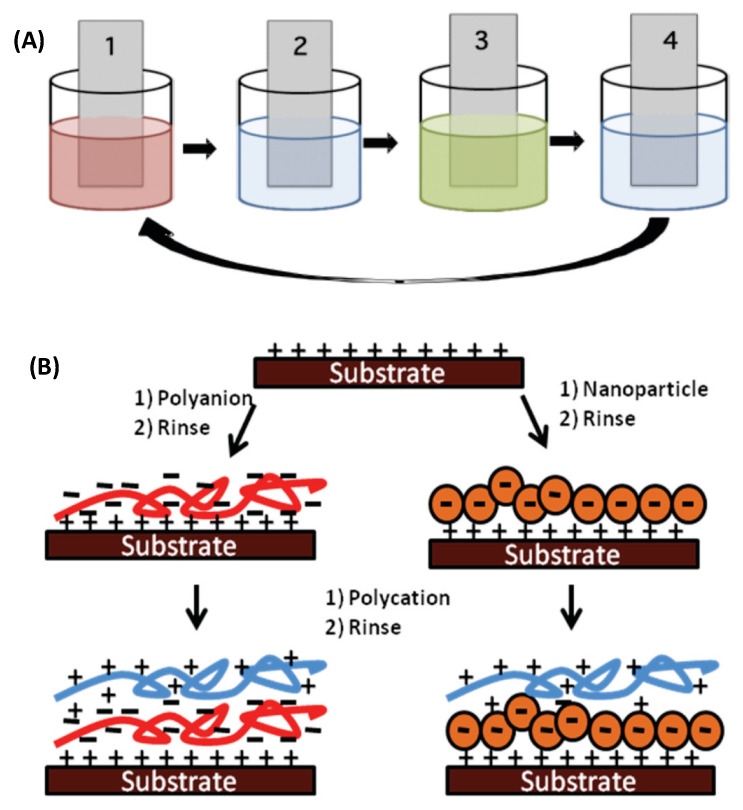
(**A**) Scheme of the LBL film deposition. Steps 1 and 3 represent the adsorption of polyanion and polycation; steps 2 and 4 are washing steps. (**B**) Two adsorption routes, depicting LBL deposition for polymers and polymers with NPs. Reproduced with permission from American Chemical Society [333].

The layer-by-layer (LbL) assembly technique has emerged as a versatile and precise manufacturing method for nacre-inspired materials, enabling the controlled fabrication of hierarchical brick-and-mortar architectures that mimic the natural arrangement of stiff inorganic platelets and soft organic interlayers. The identification of LbL-fabricated nacre-inspired structures typically involves microscopic and spectroscopic characterisation to confirm the alternating lamellar arrangement, uniform layer thickness, and interfacial bonding between phases [323,324]. Figure 13 shows an example of the composite layer-by-layer (LbL) assembly technique using inorganic nanoparticles and polymers. Mechanistically, LbL assembly relies on the sequential deposition of oppositely charged or chemically complementary building blocks—such as cationic and anionic polyelectrolytes, or inorganic nanosheets and polymer matrices—onto a substrate, driven by electrostatic attraction, hydrogen bonding, or other intermolecular forces [323,325]. This bottom-up approach allows the fine-tuning of composite composition, layer number, and sequence, thereby enabling control over mechanical performance and functional properties. In nacre-inspired systems, the inorganic phase (montmorillonite, hydroxyapatite, graphene oxide) is often deposited as nanoscale platelets, followed by organic binders (chitosan, polyvinyl alcohol, silk fibroin), creating a staggered microstructure that enhances toughness through mechanisms such as crack deflection, tablet interlocking, and energy dissipation at interfaces [324,326,327]. While LbL assembly offers a high structural precision and adaptability, challenges remain in scaling the process for industrial production due to the repetitive deposition cycles, potential porosity defects, and the need for environmentally benign solvents [322,328]. Nevertheless, its ability to replicate nacre’s hierarchical order and synergistic mechanical behaviour makes LbL assembly a key technique for developing advanced bioinspired composites. The layer-by-layer (LbL) assembly of nacre-inspired materials exhibits significant advantages, including enhanced mechanical properties such as high hardness and a reduced modulus of ~65 GPa due to strong electrostatic and hydrogen bonding between layers, functional surface characteristics like hydrophobicity, chemical resistance, and underwater superoleophobicity for oil–water separation, versatility in integrating inorganic platelets with organic polymers for multifunctional designs, and precise control over micro/nano-architecture to tune mechanical and wetting properties [282,329,330]. However, it also presents limitations, notably scalability challenges from its inherently slow sequential deposition compared to methods like freeze casting or rapid mineralisation, low porosity that restricts permeation flux in separation applications, environmental stability issues where Ca^2+^-coordination crosslinking can degrade in acidic conditions, and difficulties in incorporating hydrophobic polymers with hydrophilic clays without additional surface modification steps [103,331,332].

### 7.3. Evaporation-Induced Self-Assembly (EISA)

**Figure 14:**
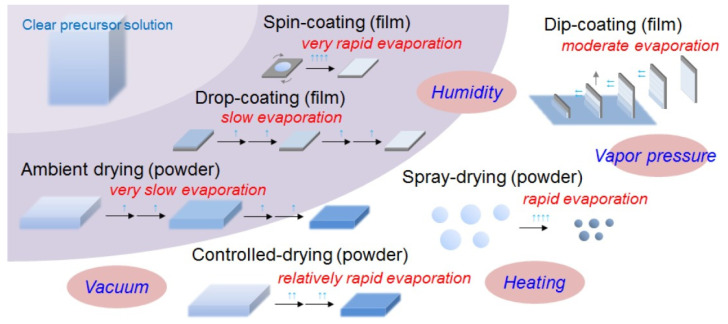
A summary of the fabrication processes for obtaining materials having surfactant-assisted mesopores designed through the EISA process. Reproduced with permission from John Wiley and Sons [337].

Evaporation-induced self-assembly (EISA) has emerged as a versatile and scalable manufacturing technique for fabricating nacre-inspired materials, enabling the creation of highly ordered, layered architectures that mimic the hierarchical structure of natural nacre [151,334]. In this approach, a homogeneous mixture of inorganic fillers (such asboron nitride nanosheets, MXene, nanoclays) and polymeric or biopolymeric matrices (chitosan, cellulose nanocrystals) is prepared, often aided by surfactants or block copolymers as structure-directing agents [151,335]. Controlled solvent evaporation (typically under mild thermal conditions) induces phase separation and self-organisation through van der Waals forces, hydrogen bonding, and electrostatic interactions, resulting in a brick-and-mortar microstructure with aligned platelets embedded in a continuous matrix [151,334,336]. Figure 14 illustrates that ordered mesoporous materials can be produced through EISA methods, where solvent evaporation is carefully controlled through humidity, vapor pressure, heating, or vacuum conditions. Films are typically made using spin, dip, or drop coating, while powders and monoliths are formed via ambient, vacuum, or spray drying [337]. The mechanism in EISA involves sequential stages [338,339]:

(1) The concentration of components at the liquid–vapour interface due to solvent loss.
(2) The nucleation and ordering of platelets or micelles into lamellar domains.
(3) Structural fixation via matrix solidification or polymer vitrification.

This molecular self-assembly approach offers several advantages: a precise control over microstructural orientation, compatibility with diverse filler–matrix systems, scalability to large-area films or bulk composites, and the ability to tailor mechanical, thermal, and electrical properties by adjusting processing parameters [151,338,340]. Moreover, EISA is relatively simple, cost-effective, and environmentally friendly, especially when using water-based systems and avoiding high-temperature processing [334,341]. However, the limitations include a sensitivity to evaporation conditions, which can affect reproducibility and uniformity [342]; challenges in achieving a precise alignment with high-inertia fillers such as h-BN [211]; and potential constraints on controlling defect density or on scaling to complex 3D geometries. Despite these drawbacks, EISA remains a promising route for engineering nacre-mimetic materials with tuneable multifunctionality, bridging bioinspired design principles with practical manufacturing scalability.

### 7.4. Vacuum Filtration/Paper-Making

In recent investigations, the vacuum filtration (paper-making) technique has been identified as a versatile method that mimics the natural brick-and-mortar structure of nacre by filtering suspensions of functionalized nanosheets or platelets to achieve densely packed, aligned layers, whose interfacial bonding is enhanced through chemical modifications such as polydopamine, tannic acid, or 2-ureido-4[1H]-pyrimidinone [343,344,345]. This method leverages the principles of traditional paper-making (disassembling, refining, and reassembling fibres) to produce composites that exhibit superior mechanical properties, such as increased tensile strength and toughness, primarily due to optimised interlayer friction and energy dissipation mechanisms [326,346,347]. Despite its advantages, including low cost, environmental benignity, and scalability in terms of film area [348,349], the technique faces inherent challenges in achieving uniform nanoscale alignment and in scaling up for complex 3D architectures compared to advanced methods like freeze casting or vacuum-assisted resin transfer moulding [101,310]. Thus, while its simplicity and economic feasibility render it attractive for eco-friendly and sustainable materials, a further integration with complementary fabrication techniques is required to overcome its limitations and fully harness its multifunctional potential.

### 7.5. Spray Coating/Doctor-Blading

Spray coating and doctor-blading are two scalable, solution-based manufacturing techniques increasingly applied in the fabrication of nacre-inspired materials due to their ability to produce layered, brick-and-mortar architectures with controlled thickness and morphology. Spray coating involves atomising a precursor suspension onto a substrate, enabling a precise control over layer deposition, adaptability to diverse material systems, and potential for large-area coverage [322,350]. In nacre-inspired composites, this method can deposit alternating inorganic and organic layers, enhancing mechanical stability, barrier properties, and functional performance. However, challenges include achieving uniform coverage, avoiding surface defects, and ensuring reproducibility, which require a careful optimisation of spray parameters and equipment design [350]. Doctor-blading (or tape casting) operates by spreading a slurry between a blade and substrate, with the film thickness determined by the blade gap and slurry rheology [351,352,353]. This technique is valued for its simplicity, low cost, and suitability for large-area coatings; in nacre-inspired systems, it facilitates platelet alignment and uniform layer lamination. It accommodates a wide range of materials, including inorganic particles with binders (e.g., PVDF in NMP) and self-solidifying polymers, without requiring intrinsic adhesion [351]. The mechanisms in both methods rely on sequential deposition and solvent evaporation to form dense, ordered lamellae that mimic natural nacre’s hierarchical structure, with doctor-blading offering a more deterministic thickness control and spray coating providing greater flexibility for complex geometries. Advantages include scalability, versatility in material choice, and compatibility with existing industrial processes [350,351,352]. Limitations involve potential non-uniformity in spray coating, difficulty in achieving nanoscale precision in doctor-blading, and the need for post-processing to enhance interfacial bonding in some nacre-like composites [350,353]. These techniques represent practical routes for translating nacre’s toughening principles into engineered materials, with doctor-blading excelling in cost-effective, uniform films and spray coating offering adaptability for functionalised, multi-material structures.

### 7.6. Sol–Gel Coating and Assembly

The sol–gel coating and assembly manufacturing technique has emerged as a versatile and effective approach for fabricating nacre-inspired materials, offering precise control over chemical composition, microstructure, and hierarchical architecture [318,354]. In this method, reactive precursors undergo hydrolysis and polycondensation to form a stable sol, which is subsequently transformed into a three-dimensional gel network. This gel can be deposited via dip coating, spin coating, or spray coating onto substrates with complex geometries, enabling conformal coverage and uniform, fine-grained structures [354]. For nacre-inspired assemblies, the sol–gel-film transformation method enables the fabrication of layered composite films that mimic the hierarchical arrangement of natural nacre, thereby enhancing mechanical strength and fracture toughness [354]. Mechanistically, the process benefits from molecular-level mixing, ensuring high homogeneity and enabling the incorporation of functional dopants, such as antimicrobial agents, nanoparticles, or bioactive phases [355,356]. The advantages of sol–gel coating in nacre-inspired materials include low processing temperatures, adaptability to diverse substrates, tuneable porosity and thickness, high purity, and the ability to integrate organic–inorganic hybrid functionalities [318,354,357]. Moreover, it is cost-effective, environmentally friendly, and capable of producing coatings with excellent adhesion, corrosion resistance, and tailored surface properties such as hydrophobicity or bioactivity [355,358]. However, the technique also presents challenges: controlling coating thickness and porosity, ensuring reproducibility across batches, and managing the complexity of multi-step processing (hydrolysis, condensation, ageing, drying, heat treatment) [359]. Additionally, prolonged ageing and drying times can slow production, and in some cases the mechanical properties of pure sol–gel-derived materials may be insufficient without reinforcement [360]. Sol–gel coating and assembly methods provide a powerful platform for engineering nacre-inspired composites, balancing structural precision and functional versatility, though the optimisation of process parameters remains critical for achieving consistent high-performance outcomes.

### 7.7. Magnetic/Field-Assisted Alignment

Magnetic or field-assisted alignment manufacturing has emerged as a powerful technique for producing nacre-inspired materials with highly ordered brick-and-mortar architectures. In this approach, ferromagnetic or paramagnetic fillers—such as alumina platelets, metallic flakes, or magnetically coated carbon nanomaterials—are dispersed in a matrix and subjected to an external magnetic field during processing, inducing their orientation along the field lines [311,361,362,363]. In magnetic field-assisted freeze casting, for example, the applied field aligns both the flakes and the walls of the cellular solid parallel to the field, while directional solidification imposes a secondary alignment along the freezing direction, yielding an orthotropic microstructure with superior stiffness, strength, and toughness compared to non-aligned counterparts [361]. Mechanistically, alignment occurs through torque exerted on anisotropic particles with magnetic susceptibility, causing a head-to-tail or planar orientation; subsequent solidification, gelation, or curing locks the aligned structure in place [364,365]. This method is integrated with additive manufacturing, in which electromagnetic solenoids or programmable magnetic fields align fillers layer-by-layer prior to photopolymerization, enabling complex geometries with controlled anisotropy [362,366].

The advantages of magnetic/field-assisted alignment include enhanced mechanical, thermal, and electrical properties due to anisotropic microstructures; scalability for large-volume production; compatibility with diverse matrices; and the ability to fine-tune filler orientation for multifunctional performance [361,363]. It is also cost-effective when implemented with permanent magnets and can be combined with other alignment strategies such as shear- or evaporation-induced ordering [363,366]. However, limitations remain: only fillers with a sufficient magnetic responsiveness can be directly aligned, necessitating magnetic coatings for inert materials like graphene or CNTs [362]; the precise control over alignment uniformity and dispersion is challenging, especially in bulk systems where gelation kinetics and particle mobility must be balanced [364]; and complex equipment or field-responsive feedstocks may be required for additive manufacturing integration [362,366]. Despite these challenges, magnetic- or field-assisted alignment remains a versatile and promising route for fabricating high-performance nacre-inspired composites with tailored anisotropy for structural, thermal management, and functional applications.

### 7.8. Hot Pressing/SPS Densification

Hot pressing and spark plasma sintering (SPS) are the primary densification techniques increasingly used in the fabrication of nacre-inspired materials, owing to their ability to achieve a high density and tailor microstructures within short processing times. In both methods, powders are compacted within a die under uniaxial pressure, but SPS distinguishes itself by using pulsed direct current to generate Joule heating either in the die or directly in the sample, enabling extremely rapid heating rates (up to 1000 °C/min) and lower sintering temperatures compared to conventional hot pressing [367,368,369]. The densification mechanism in SPS involves an initial mechanical consolidation followed by accelerated particle bonding through localised heating, grain boundary diffusion, plastic deformation, and, in some cases, liquid-phase-assisted rearrangement [370,371]. This rapid thermal cycle suppresses grain growth, preserves fine microstructures, and can purify powder surfaces by breaking oxide films, thereby facilitating strong interfacial bonding in nacre-inspired structures [372,373]. The main advantages of SPS over hot pressing include shorter dwell times, reduced energy consumption, enhanced densification of difficult-to-sinter materials, and improved control over microstructural homogeneity [374,375,376]. However, limitations remain: SPS equipment is costly, die size constraints limit large-scale production, and the process requires electrically conductive tooling, which can restrict material geometries and necessitate careful design to avoid thermal gradients [377,378]. Additionally, although SPS can rapidly achieve near-full density, residual porosity or structural defects may persist if powder purity and particle-size distribution are not optimised, potentially affecting mechanical performance in nacre-inspired composites [379]. Both hot pressing and SPS are important pathways for densifying nacre-inspired materials, with SPS offering significant processing advantages, particularly for complex, multiphase architectures where fine microstructural control is critical.

### 7.9. Coextrusion/Extrusion/Roll Compaction

Coextrusion, extrusion, and roll compaction are increasingly explored as scalable fabrication routes for nacre-inspired materials due to their ability to produce layered architectures with a controlled thickness and orientation. Coextrusion enables the parallel stacking of dissimilar polymers or composites into alternating lamellae, as demonstrated in polylactide/poly (butylene adipate-co-terephthalate) blends where interfacial diffusion—tuned by layer number—was critical to achieving a high strength–toughness balance [380]. The mechanism involves the extension–compression-coupled flow fields in the die, which align and compact layers while promoting interfacial bonding. Extrusion, including hot extrusion, can refine nacre-inspired architectures in metal–ceramic systems by reducing the layer thickness and transitioning from continuous to quasi-continuous ceramic-rich layers, thereby enhancing both strength and ductility through the improved coordination of plastic deformation and back-stress hardening [381]. Roll compaction (or roll rolling) has been applied to sinterless ceramic–polymer systems, such as hydroxyapatite–calcium carbonate/sodium alginate composites, where repeated rolling disperses the organic phase into ultrathin lamellae, improving fracture toughness via crack deflection, bifurcation, and bridging [382]. These techniques share the advantage of scalability, structural control, and compatibility with diverse material systems, making them attractive for engineering and biomedical applications [101,383]. However, they also present limitations: coextrusion and extrusion may entail high tooling costs and limited microstructural complexity relative to self-assembly methods [101,195], whereas roll compaction may be constrained by achievable layer uniformity and interfacial chemistry. Additionally, all three methods must address challenges in achieving the hierarchical multiscale features of natural nacre, which are key to its exceptional mechanical performance [327]. These deformation-based processing routes offer promising, industry-compatible pathways for producing nacre-inspired composites, but a further optimisation of interfacial engineering and hierarchical structuring is essential to fully replicate nacre’s multifunctional properties.

### 7.10. Granulation into Deformable Microspheres

Granulation into deformable microspheres represents a promising fabrication route for nacre-inspired materials, enabling the translation of nacre’s hierarchical “brick-and-mortar” architecture into scalable, processable forms. The identification of such microspheres typically relies on morphological and structural characterisation techniques—such as scanning electron microscopy (SEM) and spectroscopy—to confirm uniform size, shape, and internal lamellar organisation, as well as the presence of organic–inorganic interfaces that mimic natural nacre [384]. Mechanistically, granulation involves the controlled formation of microspheres via processes such as high-shear mixing or ultrasonic spray drying, in which precursor suspensions containing mineral platelets and polymeric binders undergo emulsification and solidification [385]. In nacre-inspired systems, this step can be coupled with in situ mineralisation or self-assembly, allowing the microspheres to retain deformability while preserving load-transfer pathways akin to tablet sliding in natural nacre [327,386]. The advantages of this technique include an improved handling and packing density, tuneable mechanical properties by controlling microsphere composition and size, and the potential for large-scale production with consistent quality [103,385]. Moreover, deformable microspheres can facilitate energy dissipation under stress, enhancing toughness without severely compromising stiffness [327,387]. However, limitations remain: granulation conditions must be carefully optimised to avoid defects or non-uniformity, and integrating microspheres into bulk nacre-inspired composites can be challenging due to interfacial bonding issues and the potential loss of long-range structural order [385,388]. Additionally, while deformability aids impact resistance, excessive softness may reduce load-bearing capacity, necessitating a balance between flexibility and strength [265,389]. Granulation into deformable microspheres shows a versatile pathway for engineering nacre-inspired materials, but its success depends on the precise control over microstructural fidelity and composite integration.

### 7.11. 3D Printing/Additive Manufacturing

Recent advances in 3D printing, or additive manufacturing (AM), have enabled the precise fabrication of nacre-inspired materials with complex hierarchical architectures that closely mimic the natural brick-and-mortar microstructure of nacre (shown in Figure 3) [101,310,390,391,392]. The identification of these materials typically involves reproducing key structural features—such as aligned platelets, wavy interfaces, and soft–hard phase alternation—using multi-material printing platforms and high-resolution techniques like fused deposition modelling (FDM), digital light processing (DLP), and two-photon polymerisation (2PP) [97,391,393]. Mechanistically, (AM) enables the control over platelet aspect ratio, interface geometry, and constituent elasticity, thereby optimising strength and toughness through crack deflection, energy dissipation, and the prevention of stress localisation [392,394]. For example, electrically assisted 3D printing can align graphene nanoplatelets within a polymer matrix, producing lightweight composites with integrated self-sensing capabilities [390], while dual-extruder systems can combine rigid PLA with flexible TPU to balance stiffness and impact resistance [97,323]. The utilisation of 3D printing technologies in the fabrication of nacre-inspired materials represents a significant advancement in composite manufacturing, primarily driven by unparalleled design freedom that facilitates the realisation of complex geometries and hierarchical layering [310,395]. This approach affords researchers precise microstructural control for the deliberate tailoring of mechanical properties [392,396], alongside multi-material capabilities that enable functional integration, such as self-sensing mechanisms and enhanced impact resistance [390,393]. While additive manufacturing offers superior potential for bulk composite scalability compared to traditional methodologies [101,397], the field faces critical technical challenges; specifically, scaling production often incurs a loss of microstructural fidelity [101,383], and material versatility remains constrained, as not all ceramics or polymers are printable with the requisite resolution [97,391]. Furthermore, process complexity—such as the risk of cross-contamination in multi-material stereolithography [393] and the inherent mechanical trade-offs between stiffness and toughness when modulating soft-phase elasticity [268]—continues to necessitate rigorous optimisation. Additive manufacturing offers a versatile and powerful route to nacre-inspired materials, enabling the integration of natural toughening mechanisms into synthetic composites for applications ranging from protective gear to structural components [394,398]. However, overcoming scalability, material compatibility, and process integration challenges remains critical for broader industrial adoption.

### 7.12. In Situ Mineralisation/Biomineralisation-Inspired

In situ mineralisation and biomineralisation-inspired techniques have emerged as powerful strategies for fabricating nacre-inspired materials, enabling the replication of the hierarchical “brick-and-mortar” architecture and physicochemical properties of natural nacre under mild conditions [310,354]. The identification of these processes often relies on advanced in situ characterisation methods, such as dual-probe fluorescence, to monitor early-stage events, including prenucleation cluster (PNC) formation, amorphous-phase aggregation, and crystallisation dynamics at the molecular and nanoscale levels [399]. Mechanistically, biomineralisation proceeds via a non-classical crystallisation pathway. PNCs form and aggregate into amorphous intermediates, which then transform into crystalline mineral phases under the guidance of organic matrices rich in functional groups (such as carboxylates, sulphates) that act as nucleation sites [400,401]. In situ mineralisation in synthetic systems mimics this by introducing mineral precursors directly into pre-formed organic frameworks such as (hydrogels or 3D-printed scaffolds), where enzymatic or polyelectrolyte additives (polyaspartic acid) enhance nucleation efficiency, control crystal morphology, and increase mineral content. This approach allows the tailoring of mechanical properties, such as toughness and stiffness, while maintaining biocompatibility, making it particularly attractive for biomedical applications like bone regeneration [402,403]. Advantages include the ability to operate under ambient conditions, a precise control over hierarchical structure, improved mechanical performance, and enhanced biological functionality [310,402]. Disadvantages involve challenges in achieving uniform mineral distribution, controlling crystallinity, and scaling up production; moreover, the complexity of biological mineralisation mechanisms means that synthetic analogues may still fall short of fully replicating natural nacre’s performance [404]. In situ mineralisation and biomineralisation-inspired methods represent a convergence of materials science and biology, showing versatile pathways to high-performance nacre-inspired composites while highlighting the need for deeper mechanistic understanding to overcome current limitations.

## 8. Conclusions

This review explores the sophisticated structure of nacre, which consists mainly of 95% aragonite calcium carbonate platelets embedded within a 5% organic biopolymer matrix. Nacre demonstrates the interplay between chemistry, material structure, and mechanisms, providing superior properties using only a modest organic–inorganic composite system. The hierarchical brick-and-mortar stacked morphology, organic interfaces, and tailored mineralisation processes collectively facilitate effective energy dissipation mechanisms and serve as a classical example for probing structure–property correlations in biological hard tissues.

Historically, the scientific journey of nacre research, beginning with Hatchett’s in 1799 classification, has evolved from a fundamental chemical curiosity into a cornerstone of modern biomimetics. Hatchett’s early distinction between “porcelaneous” and “nacreous” types provided the first evidence that life could manipulate mineral matter through an organic mediator. Research on nacre-inspired materials dates back to early biomimetic studies in the 1990s and early 2000s, which sought to replicate the natural processes of biomineralisation found in mollusc shells. Over time, this field has progressed from structural characterisation to the design of advanced artificial composites, such as ceramic–metal hybrids and alumina–cyanate resins developed in the 2020s.

Nacre-inspired materials began with first-generation lamellar ceramic–polymer assemblies and evolved towards designed, multiscale systems incorporating mineral bridges, amorphous interphases, and engineered residual stresses. These approaches have enabled biomimetic composites to match or exceed the toughness-enhancing capabilities of nacre while maintaining a high specific stiffness, thereby confirming the nacre material paradigm as a robust design choice for advanced structural materials. These innovations achieve outstanding mechanical performance through mechanisms such as platelet sliding, mineral bridging, and strain hardening.

Nacre-inspired fabrication strategies seek to replicate the characteristic “brick-and-mortar” hierarchical structure in order to optimise mechanical performance. Techniques such as freeze casting have demonstrated flexural strengths approaching 931 MPa and toughness values exceeding 50 MPa·m^1/2^, whereas layer-by-layer (LbL) assembly has yielded elastic moduli of approximately 65 GPa. Advanced processing routes, including spark plasma sintering (SPS) with heating rates reaching 1000 °C min^−1^, together with scalable approaches such as additive manufacturing, aim to emulate nature’s ≈95 vol% ceramic composition, thereby reconciling microstructural precision with large-scale manufacturability.

Looking ahead, there are numerous opportunities to integrate these nacre-inspired architectures with additional functionalities, such as thermal management, optical properties, self-healing, and environmental resistance, to create a multifunctional platform. By leveraging these bioinspirations alongside developing technologies such as fabrication, optimisation through data, and sustainable chemistry, it is anticipated that ideas inspired by nacre can be applied into practical solutions.

## 9. Nacre-Inspired Gaps and Future Direction

While nacre-inspired materials have made remarkable advances in mimicking the hierarchical “brick-and-mortar” structure of natural nacre, several fundamental scientific questions and engineering bottlenecks remain. At the fundamental level, the multiscale toughening mechanisms, including nanoasperities, mineral bridges, platelet interlocking, and viscoplastic energy dissipation in the process zone, are well-documented in natural nacre [327,405], yet their quantitative contributions and synergistic activation in synthetic analogues are not fully understood. The interaction between intrinsic toughening (plastic dissipation ahead of the crack tip) and extrinsic toughening (crack tip shielding) is highly sensitive to platelet geometry, overlap, and randomness, but predictive models remain incomplete [406,407]. Similarly, the microstructure–property relationships under extreme conditions—such as high-velocity impact, cyclic fatigue, and photothermal environments—are insufficiently mapped, limiting the design space for protective and functional applications [408,409,410]. From a technological perspective, the scalability of fabrication is a persistent challenge. Conventional methods such as layer-by-layer assembly, vacuum-assisted filtration, and ice-templating yield high-fidelity microstructures but are slow, solvent-intensive, and limited to small areas [324,411]. Even advanced additive manufacturing approaches, while enabling complex geometries and multi-material integration, face material compatibility issues—particularly in achieving strong interfacial bonding between stiff tablets and soft matrices without sacrificing flexibility or toughness [265,412,413]. Moreover, authentic biomimetic fidelity is often compromised when substituting natural microscale aragonite platelets with nanosheets (e.g., graphene, MXene, or h-BN), leading to restacking, reduced active surface area, and weaker interfacial interactions [24,411]. While this review focuses primarily on nacre-inspired structures, it is important to note that the identified challenges (strength–toughness trade-off and the complexities of multifunctional integration) are shared bottlenecks across the broader field of biomimetic structural materials. Achieving precise orientation control and hierarchical complexity remains a hurdle not only for “brick-mortar” geometries but also for other biological motifs such as Bouligand structures and osteon-mimetic systems. As noted in the recent literature, these universal challenges in high-viscosity 3D printing systems require cross-disciplinary solutions that bridge the gap between material rheology and structural biomimetics [414,415,416].

Future research should address these gaps through mechanistic quantification, developing integrated experimental–computational frameworks to isolate and measure the contributions of individual toughening mechanisms across scales to enable predictive microstructural design [327,406]. Furthermore, there is a need for advanced manufacturing innovations in scalable, energy-efficient, and environmentally friendly fabrication methods—such as high-resolution multi-material 3D/4D printing with AI-driven process control—to produce large-area, defect-minimised structures [412,413,417]. Interface engineering remains a priority, focusing on enhancing interfacial strength and toughness through tailored non-covalent and covalent bonding strategies, while optimising polymer thickness and functional group chemistry to improve load transfer and defect tolerance [24]. To ensure real-world viability, systematic long-term testing under mechanical, thermal, chemical, and photothermal stresses is required to understand degradation pathways and inform robust design for extreme-condition durability [408,409,410]. Additionally, researchers should pursue multifunctional integration, combining mechanical resilience with capabilities such as sensing, self-healing, damping, and electromagnetic shielding without compromising core performance [265,406]. Transitioning toward sustainability through bio-based systems is equally vital, utilising biodegradable, non-toxic constituents such as cellulose-based bioplastics and green processing routes to align with circular economy principles [411,418]. Ultimately, addressing these intertwined scientific and engineering challenges will require cross-disciplinary collaboration between materials scientists, mechanical engineers, computational modellers, and environmental analysts to enable nacre-inspired materials to evolve from laboratory curiosities into scalable, sustainable solutions for structural, biomedical, and protective applications.

## Figures and Tables

**Table 2:** Mechanical properties of nacre.

Property	Typical Range/Value	Notes	References
Young’s Modulus	60–90 GPa	Parallel to platelets; varies with species and hydration	[101]
Shear Modulus	~10 GPa	Measured parallel to platelets	[257]
Tensile Strength	35–220 MPa	Higher in hydrated state; species-dependent	[101,258]
Fracture Toughness	3.7–7 MPa·m^½^	Up to ~30× monolithic CaCO_3_; hydration increases values	[101,258]
Work of Fracture	1.24–1.65 kJ/m^2^	~3000× monolithic CaCO_3_; reflects energy absorption capacity	[256]
Elastic Modulus Increase from Tablet Waviness	+23%	Waviness also boosts strength (+65%) and toughness (+42%)	[227]
Bending Strength	~91 MPa (synthetic analogues)	Example from nacre-inspired composites	[259]

**Table 3:** Comparison of fabrication techniques for nacre-inspired materials: mechanics, structure advantages and limitations.

Fabrication Technique	Mechanical Data & Metrics	Advantages	Limitations
Freeze casting (ice templating)	Flexural strength up to 931 MPa; crack-growth toughness > 50 MPa·m^1/2^; anisotropic properties.	Versatile (ceramics, metals, polymers); eco-friendly; scalable; tuneable pore size/wall thickness; allows magnetic alignment.	Anisotropic (weak transverse properties); hard to replicate fine nanomorphology; low ceramic content compared to nature; high infiltration costs.
Layer-by-layer (LbL) assembly	High hardness; reduced modulus ~65 GPa; strong electrostatic/hydrogen bonding.	High structural precision; control over layer sequence/thickness; allows functional coatings (hydrophobicity).	Slow/repetitive process; scalability challenges; low porosity; environmental stability issues (acidic conditions).
Evaporation-induced self-assembly (EISA)	Tuneable mechanical, thermal, and electrical properties (via processing parameters).	Precise microstructural orientation; scalable to large areas; simple and cost-effective; compatible with diverse systems.	Sensitive to evaporation conditions (reproducibility); hard to align high-inertia fillers; defect density control.
Vacuum filtration	Increased tensile strength and toughness due to optimised interlayer friction.	Low cost; environmentally benign; scalable film area; simple process.	Difficult to achieve uniform nanoscale alignment; hard to scale for complex 3D architectures.
Spray coating/doctor-blading	Dense, ordered lamellae; controlled thickness.	Spray: large-area coverage. Doctor-blading: deterministic thickness control; low cost; simple.	Spray: Non-uniformity. Doctor-blading: Lacks nanoscale precision. Both may require post-processing.
Sol–gel coating and assembly	Enhanced strength and fracture toughness via hierarchical arrangement.	Complex geometries (conformal coverage); high purity; molecular-level mixing; low processing temps.	Control of thickness/porosity; reproducibility; complex/slow multi-step processing (ageing/drying).
MagneticfField-assisted alignment	Superior stiffness, strength, and toughness (orthotropic); highly ordered.	Contactless alignment; scalable volume; can define complex anisotropy (when combined with AM).	Requires magnetic fillers or coatings; challenging to control uniformity in bulk; equipment complexity.
Hot pressing/SPS	High density; fine microstructures; strong interfacial bonding.	SPS: Rapid heating (1000 °C/min); suppresses grain growth; clean grain boundaries.	High equipment cost (SPS); size constraints (die size); conductive tooling required; residual porosity risk.
Coextrusion/extrusion/roll compaction	Strength–toughness balance via interfacial diffusion; back-stress hardening.	Scalable; continuous processing; good for metal–ceramic or polymer blends.	High tooling costs; limited microstructural complexity compared to self-assembly; interfacial chemistry constraints.
Granulation (microspheres)	Energy dissipation via deformability; tuneable stiffness vs. impact resistance.	Improved handling/packing density; large-scale production; deformable units aid toughness.	Interfacial bonding issues in bulk integration; potential loss of long-range order; excessive softness reduces load capacity.
3D printing/additive manufacturing	Optimised strength/toughness via geometry; integrated self-sensing/impact resistance.	Unparalleled design freedom (complex geometries); multi-material capabilities; precise control.	Loss of microstructural fidelity at scale; material constraints (resolution limits); slow compared to bulk methods.
In situ mineralisation	Tailored toughness/stiffness; biocompatible; mimics natural nacre formation.	Ambient processing conditions; precise hierarchical control; enhanced biological functionality.	Difficult to ensure uniform mineral distribution and crystallinity; complexity of biological mechanisms.

## Data Availability

No new data were created or analysed in this study. Data sharing is not applicable to this article.
